# FunOrder: A robust and semi-automated method for the identification of essential biosynthetic genes through computational molecular co-evolution

**DOI:** 10.1371/journal.pcbi.1009372

**Published:** 2021-09-27

**Authors:** Gabriel A. Vignolle, Denise Schaffer, Leopold Zehetner, Robert L. Mach, Astrid R. Mach-Aigner, Christian Derntl

**Affiliations:** Institute of Chemical, Environmental and Bioscience Engineering, TU Wien, Vienna, Austria; Northeastern University, UNITED STATES

## Abstract

Secondary metabolites (SMs) are a vast group of compounds with different structures and properties that have been utilized as drugs, food additives, dyes, and as monomers for novel plastics. In many cases, the biosynthesis of SMs is catalysed by enzymes whose corresponding genes are co-localized in the genome in biosynthetic gene clusters (BGCs). Notably, BGCs may contain so-called gap genes, that are not involved in the biosynthesis of the SM. Current genome mining tools can identify BGCs, but they have problems with distinguishing essential genes from gap genes. This can and must be done by expensive, laborious, and time-consuming comparative genomic approaches or transcriptome analyses. In this study, we developed a method that allows semi-automated identification of essential genes in a BGC based on co-evolution analysis. To this end, the protein sequences of a BGC are blasted against a suitable proteome database. For each protein, a phylogenetic tree is created. The trees are compared by treeKO to detect co-evolution. The results of this comparison are visualized in different output formats, which are compared visually. Our results suggest that co-evolution is commonly occurring within BGCs, albeit not all, and that especially those genes that encode for enzymes of the biosynthetic pathway are co-evolutionary linked and can be identified with FunOrder. In light of the growing number of genomic data available, this will contribute to the studies of BGCs in native hosts and facilitate heterologous expression in other organisms with the aim of the discovery of novel SMs.

## Author summary

The discovery and description of novel fungal secondary metabolites promises novel antibiotics, pharmaceuticals, and other useful compounds. A way to identify novel secondary metabolites is to express the corresponding genes in a suitable expression host. Consequently, a detailed knowledge or an accurate prediction of these genes is necessary. In fungi, the genes are co-localized in so-called biosynthetic gene clusters. Notably, the clusters may also contain genes that are not necessary for the biosynthesis of the secondary metabolites, so-called gap genes. We developed a method to detect co-evolved genes within the clusters and demonstrated that essential genes are co-evolving and can thus be differentiated from the gap genes. This adds an additional layer of information, which can support researchers with their decisions on which genes to study and express for the discovery of novel secondary metabolites.

This is a *PLOS Computational Biology* Methods paper.

## Introduction

Secondary metabolites (SMs) are a diverse group of compounds with a plethora of different chemical structures and properties which are found in all domains of life, but are predominantly studied in bacteria, fungi, and plants [1]. SMs are not necessary for the basic survival and growth of an organism but can be beneficial under certain conditions. For example, pigments help to sustain radiation, antibiotics help in competitive situations, and toxins can serve as defensive compounds or as virulence factors [2,3]. Notably, many SMs are used by humankind as drugs and pharmaceuticals, pigments and dyes, sweeteners and flavours, and most recently also as precursors for the synthesis of plastics [4]. The study of the secondary metabolism holds the promise for novel antibiotics, pharmaceuticals and other useful compounds [5].

A major hinderance in the discovery of yet undescribed SMs is the fact that most SMs are not produced under standard laboratory conditions, as they do not serve a purpose for the organisms then. Currently, different strategies are followed to circumvent this problem [6,7]. Untargeted approaches aim to induce the expression of any SM. To this end, biotic and abiotic stresses are applied, or global regulators and regulatory mechanisms are manipulated [8]. These strategies may lead to the discovery of novel compounds, whose corresponding genes have to be identified subsequently by time-consuming and expensive methods [7]. An extreme example are the aflatoxins, major food contaminants with serious toxicological effects [9]. It took over 40 years from the discovery of the aflatoxins as the causal agent of “turkey X” disease in the 1950s [10] until the corresponding genes were finally described in 1995 [11]. Targeted SM discovery approaches aim to induce the production of specific SMs by either overexpressing genes in the native host or by heterologous expression in another organism [12]. The targeted approaches, also called reverse strategy or bottom-up strategy allows a direct connection of SMs to the corresponding genes and does not rely on the inducibility of SM production in the native host. Inherently, the bottom-up approach is depending on modern genomics and accurate gene prediction tools [13].

**Fig 1:**
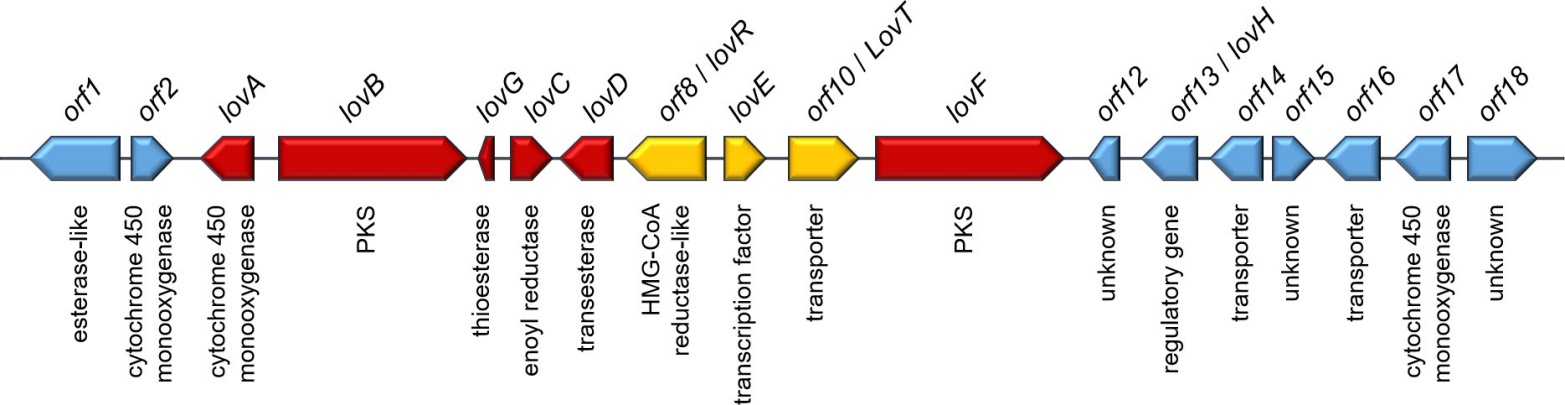
Schematic representation of the lovastatin BGC from *Aspergillus terreus* (lov). In red the biosynthetic genes for SM production, in gold the further essential genes, and in blue the genes not involved in the biosynthetic pathway.

In bacteria and fungi, the genes responsible for the biosynthesis of a certain SM are often co-localized in the genome, forming so called biosynthetic gene clusters (BGCs) [14,15]. The BGCs consists of one or more core genes, several tailoring enzymes, and genes involved in regulation and transport. As all these genes are essential for the production of a SM in the native host, we will refer to them as “essential genes” in this study. The core genes are responsible for assembling the basic chemical scaffold, which is further modified by the tailoring enzymes yielding the final SM [16]. We refer to the core genes and the tailoring genes as “biosynthetic genes” in this study. Depending on the class of the produced SM, the core genes differ. In fungi, the main SM classes are polyketides (e.g. the cholesterol-lowering drug lovastatin [17] and the mycotoxin aflatoxin [9]) and non-ribosomal peptides (e.g. the immunosuppressant cyclosporine [18] and the antibiotic penicillin [19]), with polyketide synthases (PKS) or non-ribosomal peptide synthetases (NRPS) as core enzymes, respectively. Other SM classes are terpenoids, alkaloids, melanins [20,21], and ribosomally synthesized and posttranslationally modified peptides (RiPPs) [22,23], whose corresponding genes may also be organized in BGCs. As mentioned, BGCs may also contain genes encoding for transporters [24], transcription factors [25], or resistance genes [26]. While their gene products are not directly involved in the biosynthesis of a SM they are still essential for the biosynthesis; we will call them „further essential genes”in the following and differentiate them from the „biosynthetic genes“. The biosynthetic genes and the further essential genes are both necessary for the biosynthesis of a SM in the native organisms. In contrast, only the biosynthetic genes and a selection of the further essential genes (e.g. transporters) are necessary for heterologous expression [reviewed in [27]]. Notably, fungal BGCs often also contain genes that are not necessary for the production of a SM, the so-called gap genes. The gap genes are not involved in the biosynthesis, regulation, or transport of the SM, but have an unrelated function (Fig 1). We would like to stress here, that this cannot be predicted based only on the class of the gene product. For instance, a gene encoding for a transporter in the aflatoxin BGC was reported to have no significant role in aflatoxin secretion [28].

As mentioned, the bottom-up approach for SM discovery is depending on modern genomics and the accurate prediction of genes and BGCs. Each important gene missing in the prediction is detrimental for obvious reasons, whereas each unnecessarily considered gap gene makes the study of a BGC more complicated and complex, and the construction and transformation processes for heterologous expression more challenging. Currently, several BGC prediction tools are available for fungi. Some tools for genome mining are antiSMASH [29], CASSIS and SMIPS [30], SMURF [31], TOUCAN, a supervised learning framework capable of predicting BGCs on amino acid sequences [32], and DeepBGC, an unrestricted machine learning approach using deep neural networks [33]. These tools are effective and successful in finding and predicting BGCs based solely on genomic data. AntiSMASH uses a rule-based approach to identify BGCs based on the identification of core or signature enzymes and applies a greedy approach to extend a cluster on either side. This may result in overlaps or combinations of closely situated clusters. However, the genes within the predicted BGCs are classified into core biosynthetic genes, additional biosynthetic genes, transport-related genes, regulatory genes, and other genes based on profile hidden Markov models by the antiSMASH tool. The BGC prediction method of CASSIS and SMIPS is based on the principle that the promoter regions of genes in a BGC contain one or more shared motif, as they are co-expressed and presumably regulated by the same regulatory factors and/or mechanisms [30].

As mentioned above, the class of an enzyme may be a good indication for a potential involvement in the biosynthesis of a SM but does not guarantee a correct prediction. This problem can be solved by the analysis of transcriptome data because the genes necessary for SM production within a BGC are normally co-expressed with each other but not with the gap genes [34]. Notably, this demands the knowledge of expression conditions and does not work for silent BGCs. However, it is an obvious advantage to have as much information as possible about a BGC before studying it in the native host or performing heterologous expression for a bottom-up approach for SM discovery.

We speculate that a comparative genomics analysis focusing on the evolutionary history of the genes in a BGC might be a feasible alternative to a transcriptomics analysis in fungi for the following reasons. In general, BGCs are suggested to undergo a distinct and faster evolution than the rest of the genome, based on different mechanisms and genetic drivers [16,35–40]. In bacteria, the evolution of BGCs is strongly influenced by the strong occurrence of horizontal gene transfer in these group of microorganism [39]. Medema et al. performed a large-scale computational analysis of bacterial BGCs and found that many BGCs consist of sub-clusters. These sub-clusters encode for enzymes that work together to form a distinct chemical structure. Notably, this sub-clusters were described as “independent evolutionary entities” and the contained genes are co-evolving. The authors suggested a “bricks and mortar” model. Therein, different sub-clusters, the “bricks” form different chemical building blocks for a secondary metabolite. Additional genes within the BGCs are encoding for enzymes that combine the building blocks, and fulfil other functions such as tailoring, regulation and transport. These individual genes are the “mortar” in the “brick and mortar” model [40]. The “bricks” correspond to what we term “biosynthetic genes” and the “mortar” to our “further essential genes”. Through horizontal gene transfer, the “bricks” can be easily exchanged and recombined to form novel BGCs and secondary metabolites[40]. Notably, not all bacterial BGCs are composed of exchangeable sub-units but some BGCs keep a stable architecture over a long time [40].

In fungi, three molecular evolutionary processes were suggested to be responsible for shaping the BGCs in a recent study, i.e., functional divergence, horizontal gene transfer, and *de novo* assembly [41]. Rokas et al. define functional divergence as a “process by which homologous BGCs, through the accumulation of genetic changes, gradually diverge in their functions changes” [41] and horizontal gene transfer as a “process by which an entire BGC from the genome of one organism is transferred and stably integrated into the genome of another through non-reproduction related mechanisms” [41]. This implies in both cases, that fungal BGCs are staying intact. Further, the genes are suggested to undergo a co-evolution which is faster than the rest of the genome [41]. Medema’s “brick and mortar” model would more or less correspond to what Rokas et al. describe as “*de novo* assembly”. This is defined as a “process by which an entire BGC is evolutionarily assembled through the recruitment and relocation of native genes, duplicates of native genes, and horizontally acquired genes” [41]. Notably, Rokas et al. state that this is the”least well-documented evolutionary process involved in the generation of fungal chemodiversity” [41], suggesting that in known and described fungal BGCs functional divergence and horizontal gene transfer are the two main evolutionary process, during which BGCs are staying intact and genes undergo a similar evolution. Further, we hypothesize that especially the biosynthetic genes in a BGC are co-evolutionary linked by the selection pressure to keep the biosynthetic pathway intact. Notably, a co-evolution analysis is a laborious and time-consuming task because a phylogenetic tree has to be calculated for each gene and then the trees compared to each other manually [42]. Recently, a method for the detection of co-evolution in bacterial BGCs was developed with the aim to identify sub-clusters [43]. That method is based on the detection of orthologous genes that are present in close vicinity in many BGCs. This method is working unsupervised but requires a large set of BGCs as input [43].

In this study we describe a method (FunOrder) that allows a fast, semi-automated co-evolution analysis using individual BGCs as input. Based on this analysis and the assumption that the essential genes undergo a shared or similar evolution, FunOrder aims to identify essential genes in BGCs. To this end, we constructed a database of fungal proteomes as basis for the identification of co-evolutionary linked genes in ascomycetes. We determine the thresholds for the detection of co-evolution within different control gene sets. Then, we evaluated FunOrder and tested the underlying hypothesis, whether essential genes within a BGC could be identified based on the principle of co-evolution. We demonstrated the robustness and the applicability of the FunOrder method by analysing different control gene sets, including empirically validated BGCs and evaluated our method using stringent statistical tests.

## Material and methods

### Construction of a fungal proteome database

In this study we aim to identify co-evolutionary linked genes in ascomycetes. As the basis for the detection of co-evolution is a suitable database [42], we compiled an empirically optimized database consisting of 134 fungal proteomes from mainly ascomycetes and from two basidiomycetes for this method (Table 1). The two basidiomycete proteomes were included for the off chance of analysing gene clusters that do not originate from ascomycetes. The database covers the complete ascomycetes phylum and was iteratively tested and optimized for the detection of co-evolution in ascomycetes. The sequences were downloaded from the National Center for Biotechnology Information (NCBI) database and the Joint Genome Institute (JGI) [44]. A short identifier, unique in the database for each proteome, was introduced to enable multiple pairwise tree comparisons by the treeKO application [45]. A custom Perl script was used for removing duplicated entries in the database. The database is deposited in the GitHub repository https://github.com/gvignolle/FunOrder (doi:10.5281/zenodo.5118984).

**Table 1:** Fungal proteomes included in the empirically optimized database.

Organism	Source Database	Identifier	Reference
*Acremonium chrysogenum*	JGI	AcCh	[46]
*Alternaria alternata*	NCBI	AlAl	[47]
*Alternaria arborescens*	NCBI	AlAr	[48]
*Alternaria gaisen*	NCBI	AlGa	[49]
*Alternaria sp*. MG1	NCBI	AlSp	[50]
*Alternaria tenuissima*	NCBI	AlTe	[49]
*Amanita muscaria*	NCBI	AmMu	[51]
*Amorphotheca resinae*	JGI	AmRe	[52]
*Arthrobotrys oligospora*	JGI	ArOl	[53]
*Arthroderma benhamiae*	JGI	ArBe	[54]
*Ascobolus immersus*	JGI	AsIm	[55]
*Aspergillus costaricaensis*	NCBI	AsCo	[56]
*Aspergillus fijiensis*	NCBI	AsFi	[56]
*Aspergillus flavus*	NCBI	AsFl	[57]
*Aspergillus fumigatus*	NCBI	AsFu	[58]
*Aspergillus homomorphus*	NCBI	AsHo	[56]
*Aspergillus ibericus*	NCBI	AsIb	[56]
*Aspergillus japonicus*	NCBI	AsJa	[56]
*Aspergillus niger*	NCBI	AsNi	[59]
*Aspergillus oryzae*	NCBI	AsOr	[60]
*Aspergillus phoenicis*	NCBI	AsPh	[61]
*Aspergillus terreus*	NCBI	AsTe	[62]
*Blumeria graminis*	JGI	BlGr	[63]
*Botryosphaeria dothidea*	JGI	BoDo	[64]
*Botrytis cinerea*	NCBI	BoCi	[65]
*Botrytis elliptica*	NCBI	BoEl	[66]
*Botrytis galanthina*	NCBI	BoGa	[66]
*Botrytis hyacinthi*	NCBI	BoHy	[66]
*Botrytis paeoniae*	NCBI	BoPa	[66]
*Botrytis porri*	NCBI	BoPo	[66]
*Botrytis tulipae*	NCBI	BoTu	[66]
*Cadophora sp*.	JGI	CaSp	[67]
*Capronia semiimmersa*	JGI	CaSe	[68]
*Chaetomium globosum*	JGI	ChGl	[69]
*Choiromyces venosus*	JGI	ChVe	[55]
*Cladonia grayi*	JGI	ClGr	[70]
*Cladophialophora bantiana*	JGI	ClBa	[68]
*Cladophialophora carrionii*	JGI	ClCa	[68]
*Cladophialophora immunda*	JGI	ClIm	[68]
*Cochliobolus heterostrophus*	JGI	CoHe	[71]
*Cochliobolus victoriae*	JGI	CoVi	[72]
*Colletotrichum nymphaeae*	JGI	CoNy	[73]
*Colletotrichum orchidophilum*	JGI	CoOr	[74]
*Colletotrichum salicis*	JGI	CoSa	[73]
*Colletotrichum simmondsii*	JGI	CoSi	[73]
*Colletotrichum tofieldiae*	JGI	CoTo	[75]
*Coniosporium apollinis*	JGI	CoAp	[68]
*Coniosporium apollinis* CBS 100218	JGI	Capo	[68]
*Corynespora cassiicola*	JGI	CoCa	[76]
*Daldinia eschscholzii*	JGI	DaEs	[77]
*Diaporthe ampelina*	JGI	DiAm	[78]
*Diplodia seriata*	JGI	DiSe	[78]
*Erysiphe necator*	JGI	ErNe	[79]
*Eutypa lata*	NCBI	EuLa	[80]
*Exophiala aquamarina*	JGI	ExAq	[68]
*Exophiala dermatitidis*	JGI	ExDe	[68]
*Exophiala oligosperma*	JGI	ExOl	[68]
*Exophiala spinifera*	JGI	ExSp	[68]
*Exophiala xenobiotica*	JGI	ExXe	[68]
*Fonsecaea monophora*	JGI	FoMo	[81]
*Fusarium fujikuroi*	NCBI	FuFu	[82]
*Fusarium graminearum*	NCBI	FuGr	[83]
*Fusarium oxysporum*	NCBI	FuOx	[84]
*Fusarium proliferatum*	NCBI	FuPr	[85]
*Fusarium pseudograminearum*	NCBI	FuPs	[86]
*Fusarium verticillioides*	NCBI	FuVe	[83]
*Gaeumannomyces graminis*	JGI	GaGr	[87]
*Glonium stellatum*	JGI	GlSt	[88]
*Hypoxylon sp*. EC38	JGI	HyEC	[77]
*Hypoxylon sp*.CO27	JGI	Hysp	[77]
*Magnaporthe grisea*	JGI	MaGr	[89]
*Magnaporthiopsis poae*	JGI	MaPo	[87]
*Meliniomyces bicolor*	JGI	MeBi	[52]
*Meliniomyces variabilis*	JGI	MeVa	[52]
*Metarhizium acridum*	NCBI	MeAc	[90]
*Metarhizium album*	NCBI	MeAl	[91]
*Metarhizium anisopliae*	NCBI	MeAn	[91]
*Metarhizium brunneum*	NCBI	MeBr	[91]
*Metarhizium guizhouense*	NCBI	MeGu	[91]
*Metarhizium majus*	NCBI	MeMa	[91]
*Metarhizium rileyi*	NCBI	MeRi	[92]
*Metarhizium robertsii*	NCBI	MeRo	[90]
*Monacrosporium haptotylum*	JGI	MoHa	[93]
*Morchella importuna*	JGI	MoIm	[94]
*[Nectria] haematococca*	NCBI	NeHa	[95]
*Nectria haematococca*	JGI	NeHa	[95]
*Neurospora crassa*	JGI	NeCr2	[96]
*Neurospora crassa* FGSC	JGI	NeCr	[97]
*Neurospora tetrasperma*	JGI	NeTe	[98]
*Oidiodendron maius*	JGI	OiMa	[51]
*Ophiostoma piceae*	JGI	OpPi	[99]
*Paecilomyces variotii*	JGI	PaVa	[100]
*Panaeolus cyanescens*	NCBI	PaCy	[101]
*Paracoccidioides brasiliensis*	JGI	PaBr	[102]
*Penicillium camemberti*	NCBI	PeCa	[103]
*Penicillium chrysogenum*	NCBI	PeCh	[104]
*Penicillium digitatum*	NCBI	PeDi	[105]
*Penicillium expansum*	NCBI	PeEx	[106]
*Penicillium nalgiovense*	NCBI	PeNa	[107]
*Penicillium oxalicum*	NCBI	PeOx	[108]
*Penicillium roqueforti*	NCBI	PeRo	[103]
*Penicillium rubens Wisconsin*	NCBI	PeRu	[109]
*Penicillium vulpinum*	JGI	PeVu	[107]
*Periconia macrospinosa*	JGI	PeMa	[67]
*Pestalotiopsis fici*	NCBI	PeFi	[110]
*Phaeoacremonium aleophilum*	JGI	PhAl	[111]
*Phaeomoniella chlamydospora*	JGI	PhCh	[78]
*Phialocephala scopiformis*	JGI	PhSc	[112]
*Pneumocystis jirovecii*	JGI	PnJi	[113]
*Pseudogymnoascus destructans*	JGI	PsDe	[114]
*Pseudomassariella vexata*	JGI	PsVe	[115]
*Rhizoctonia solani*	NCBI	RhSo	[116]
*Saccharomyces arboricola*	NCBI	SaAr	[117]
*Saccharomyces cerevisiae*	NCBI	SaCe	[118]
*Terfezia boudieri*	JGI	TeBo	[55]
*Tolypocladium ophioglossoides*	NCBI	ToOp	[119]
*Tolypocladium paradoxum*	NCBI	ToPa	[120]
*Trichoderma arundinaceum*	NCBI	TrAr	[121]
*Trichoderma asperellum*	NCBI	TrAs	[122]
*Trichoderma atroviride*	NCBI	TrAt	[123]
*Trichoderma citrinoviride*	NCBI	TrCi	[122]
*Trichoderma harzianum*	NCBI	TrHa	[124]
*Trichoderma longibrachiatum*	NCBI	TrLo	[125]
*Trichoderma reesei*	NCBI	TrRe	[126]
*Trichoderma virens*	NCBI	TrVi	[123]
*Trichophyton rubrum*	JGI	TrRu	[127]
*Tuber aestivum var*. *urcinatum*	JGI	TuAe	[55]
*Tuber magnatum*	JGI	TuMa	[55]
*Venturia inaequalis*	JGI	VeIn	[128]
*Verruconis gallopava*	JGI	VeGa	[68]
*Verticillium dahliae*	JGI	VeDa	[129]
*Xylona heveae*	JGI	XyHe	[130]
*Zymoseptoria brevis*	JGI	ZyBr	[131]
*Zymoseptoria pseudotritici*	JGI	ZyPs	[132]

The sequences were downloaded from the National Center for Biotechnology Information (NCBI) database or the Joint Genome Institute (JGI). The identifiers were used in the FunOrder software package.

### Workflow

**Fig 2:**
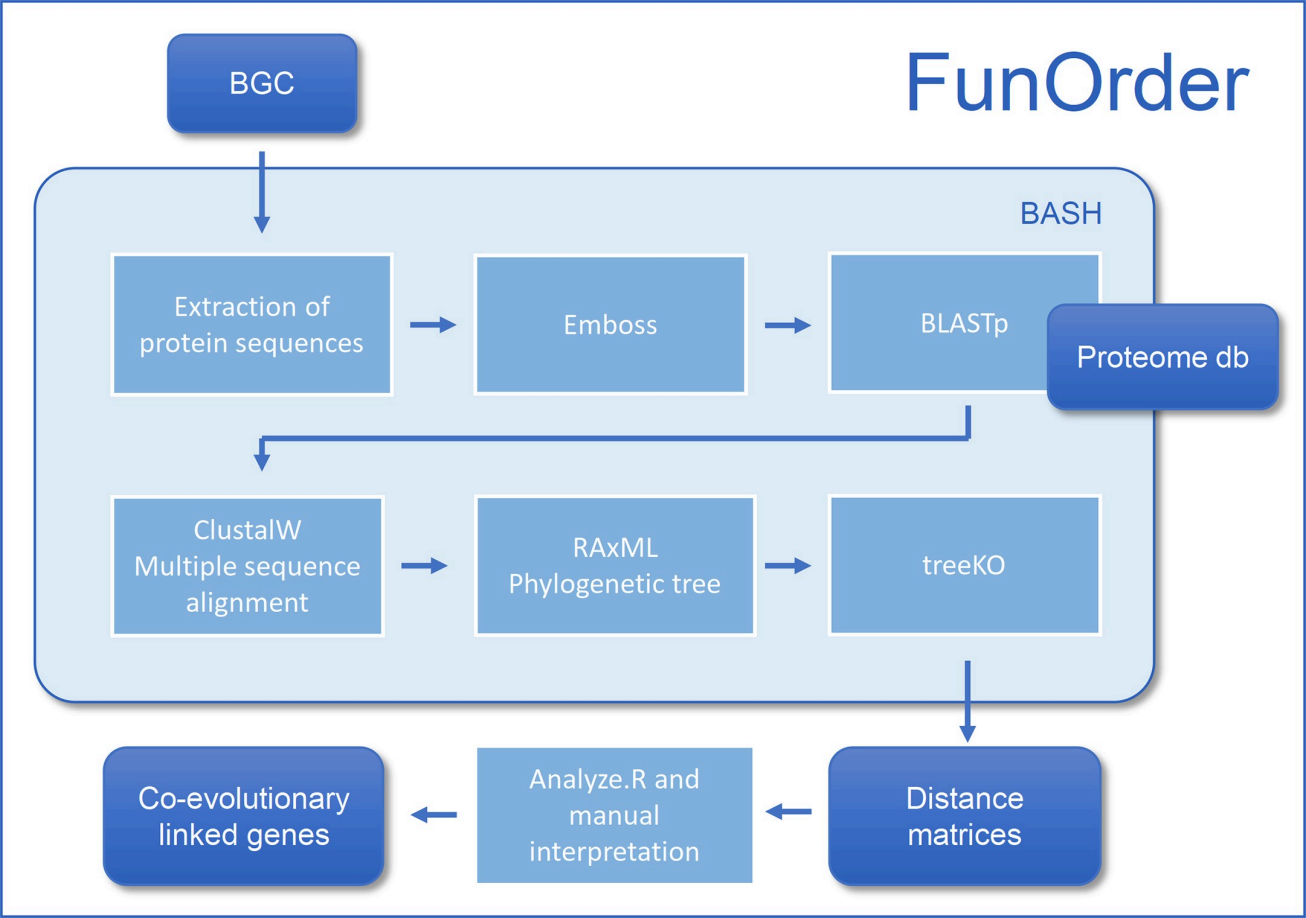
Schematic representation of the workflow of FunOrder.

The workflow for the FunOrder method is depicted in Fig 2. First, the sequences of the BGC to be analysed are fed into the software bundle. FunOrder accepts a single file in either genbank file format or fasta format as input. The input files contain BGCs predicted by tools such as antiSMASH [29] or DeepBGC [33]. In case a genbank file is provided, a python script (Genbank to FASTA by Cedar McKay and Gabrielle Rocap, University of Washington) is called to extract the amino acid sequence of the genes in the BGC and create a fasta file. The multi-fasta file is then split into individual fasta files each containing a single protein sequence. These are placed in a subfolder created for the analysis of the BGC. Each file is named either after the position of the gene in the BGC or after the respective protein sequence description. This varies from the input file and the varying annotations used (If needed this can be changed in the script following the instructions of Genbank to FASTA by Cedar McKay and Gabrielle Rocap, University of Washington). Each header of the query sequences is tagged with the identifier "query" at the beginning of the header. The individual sequences are compared to the empirically optimized proteome database (Table 1) by a sequence similarity search using blastp 2.8.1+ (Protein-Protein BLAST) [133]. The output of this search is saved in a file with the ".tab" extension. Additionally, an optional remote search of the non-redundant National Center for Biotechnology Information (NCBI) protein database can be performed, yielding a file with the "ncbi.tab" extension. This allows a preliminary manual analysis of the input sequences and facilitates subsequent annotations of the BGCs.

Next, the top 20 results of the blastp analysis are extracted and combined with the query sequence for each gene. A custom Perl script removes potential duplicate entries based on sequence identity. Using emma, a multiple sequence alignment of these protein sequences is calculated based on the ClustalW [134] algorithm, and a dendrogram computed. Based on the multiple sequence alignment, 100 rapid Bootstraps and a subsequent search for the best-scoring maximum likelihood (ML) tree are performed using RAxML (Randomized Axelerated Maximum Likelihood) [135]. The phylogenetic trees are computed using the LG amino acid substitution model. Furthermore, a standard ascertainment bias correction by Paul O. Lewis is performed. At this stage, we have obtained a phylogenetic tree (within the context of our empirically optimized database) for each protein of the input BGC.

To estimate if and to what extent the different genes within a BGC are co-evolved, the strict distance and speciation distance among the ML trees of the individual genes are calculated using the TreeKO algorithm [45]. This tool was designed for automated tree comparison and was already suggested to be used for the detection of co-evolution in protein families [45]. The tool compares the topology of different trees; a distance of 0 in both distance measures represents identical trees. In this context, a higher similarity between the different trees of the individual genes points towards a shared evolution. The strict distance is a weighted Robinson-Foulds (RF) distance measure that penalizes dissimilarities in evolutionarily important events such as gene losses and gene duplications; it has been suggested to be more significant in the detection of co-evolution than the evolutionary distance [45]. In contrast, the evolutionary or speciation distance is computed without taking evolutionary exceptions, such as duplication events or different species content of the two compared trees into account and infers shared "speciation history" based solely on topology without considering branch lengths and only considering shared species of the compared trees. Therefore, an evolutionary distance of 0 does not necessarily describe identical trees but shared "speciation history" of shared species. All pairwise strict and evolutionary distances are combined into matrices which are used as input for an R script [136–140].

In this R-script, first, the strict and evolutionary distances are summed up to a third combined distance matrix combining the information about co-evolution and shared speciation into a single measure. In our experience, this measure can be helpful to detect genes that share little co-evolution with the core-enzymes but are still essential for the biosynthesis, which is reflected in a shared speciation. The evolutionary distance is not directly part of the output of FunOrder as is not intended to be used for the detection of co-evolution. Second, the strict and the combined distance matrices are visualized as heatmaps with a dendrogram computed with the complete linkage method, to find similar clusters in these data sets. Next, the Euclidean distance within the matrices is computed and clustered using Ward’s minimum variance method aiming at finding compact spherical clusters, with the implemented squaring of the dissimilarities before cluster updating, for the two distance matrices separately, with scaled input data [141]. Lastly, a principal component analysis (PCA) is performed on the two distance matrices and the score plot of the first two principal components visualized, respectively. These outputs enable the adoption of a larger view on the distance measures and thereby allow the analysis of co-evolution within the BGC from different perspectives. We describe in a following subchapter how to interpret these visualisations.

The software bundle is written in the BASH (Bourn Again Shell) environment and includes all necessary subprograms. As BASH is the default shell-language of all Linux distributions and MacOS, FunOrder can run on these two operation systems. The FunOrder software package is deposited in the GitHub repository https://github.com/gvignolle/FunOrder (doi:10.5281/zenodo.5118984). Notably, the software package includes scripts adapted to the use on servers and for the integration in various pipelines; details on these can be found in the ReadMe file on the GitHub repository. FunOrder requires some dependencies e.g., RAxML (Randomized Axelerated Maximum Likelihood) [135] and the EMBOSS (The European Molecular Biology Open Software Suite) package [142], for details and links to all dependencies please refer to the ReadMe file on the GitHub repository.

### Compilation of benchmark gene clusters (GCs)

To test and evaluate the applicability of the FunOrder method, we used different control and test gene (or protein) sets. The sequences of all test and control sets are deposited in the GitHub repository https://github.com/gvignolle/FunOrder (doi: 10.5281/zenodo.5118984). The first set of negative control gene clusters (GCs) were 42 completely randomly generated synthetic GCs, which were created with a custom BASH script. Therein, ATGC strings of random composition and length were translated to amino acid strings using transeq from the EMBOSS package and the asterisks were removed. The second set of negative controls were 60 random GCs which were created by subsampling randomly the fungal proteome database with a Perl script from the MEME suit [143]. For each random GC a different seed number was given to guarantee non repetitive GCs, each random GC contained 3–10 randomly chosen protein sequences in a random order. These negative control GCs were subsampled from different genomes to maximize the randomness and use gene clusters that should not contain co-evolved genes.

We used a set of 30 empirically well characterized BGCs from a broad range of different genera (Table 2) as positive controls. The BGC sequences were downloaded from NCBI or the MIBiG (Minimum information about a biosynthetic gene cluster) database [144]. The sequences are available at the GitHub repository https://github.com/gvignolle/FunOrder (doi:10.5281/zenodo.5118984). All BGCs were manually inspected for correctness and completeness based on the respective literature (S1 Table, references in Table 2). We further added 2 genes on each side of the BGC to mimic the greedy gain performed by antiSMASH, if possible (sequences available) and applicable (only few or no gap genes present). Next, we defined the class of each gene (biosynthetic gene, further essential gene, gap, or extra gene) according to the described function of the enzymes in the literature (S1 Table).

**Table 2:** Empirically characterized biosynthetic gene clusters used as positive controls.

Product—BGC	Organism	MIBiG id	Reference(s)
2-Pyridon-Desmethylbassianin (dmb)	*Beauveria bassiana*	BGC0001136	[145]
Aflatoxin (afl)	*Aspergillus flavus*	BGC0000008	[146,147]
Botrydial (bot)	*Botrytis cinera*	BGC0000631	[148,149]
Cephalosporin (cef)	*Acremonium chrysogenum*	BGC0000317	[150]
Compactin (mlc)	*Penicillium citrinum*	BGC0000039	[151,152]
Cyclosporin (cyc2)	*Beauveria felina*	BGC0001565	[18,153–155]
Destruxin (dtxs)	*Metarhizium robertsii*	BGC0000337	[156]
Fumagillin (fma)	*Aspergillus fumigatus*	BGC0001067	[157]
Fumitremorgin (ftm)	*Aspergillus fumigatus*	-	[158–161]
Fumonisin (fum1)	*Fusarium oxysporum*	BGC0000063	[162]
Fumonisin (fum2)	*Fusarium verticilloides*	BGC0000062	[163–170]
Fusaric acid (FUB)	*Fusarium fujikuroi*	-	[171]
Ilicicolin H (ili)	*Neonectaria sp*. DH2	BGC0002035	[172]
Leporin (lep)	*Aspergillus flavus*	BGC0001445	[173]
Lovastatin (lov)	*Aspergillus terreus*	-	[17,62,174]
Mycophenolic acid (mpa1)	*Penicillium brevicompactum*	BGC0000104	[175–180]
Mycophenolic acid (mpa2)	*Penicillium roqueforti*	BGC0001360	[181]
Mycophenolic acid (mpa3)	*Penicillium roqueforti*	BGC0001677	[182]
Paxillin (pax)	*Penicillium paxilli*	BGC0001082	[183]
Penicillin (pen1)	*Penicillium chrysogenum*	BGC0000404	[184]
Penicillin (pen2)	*Penicillium chrysogenum*	BGC0000405	[19]
Pestheic acid (pta)	*Pestalotiopsis fici*	BGC0000121	[185]
Pneumocandin (GL)	*Glaera Iozoyensis*	BGC0001035	[186–188]
Sorbicillinol (sor1)	*Penicillium rubens*	BGC0001404	[189,190]
Sorbicillinol (sor2)	*Trichoderma reesei*	-	[191]
Tenellin (ten)	*Beauveria bassiana*	BGC0001049	[192,193]
Terrein (ter)	*Aspergillus terreus*	BGC0000161	[194]
Tetramic acid (tas)	*Hapsidospora irregularis*	-	[195]
Ustiloxin B (ust)	*Aspergillus flavus*	-	[196]
Xanthocillin (xan)	*Aspergillus fumigatus*	BGC0001990	[197]

Further, we compiled 10 protein sets containing the sequences of enzymes of conserved metabolic pathways from organisms that were not included in the proteome database, termed „Biosynthetic_pathways“, or „BioPath”(S2 Table; sequences deposited at the GitHub repository https://github.com/gvignolle/FunOrder (doi:10.5281/zenodo.5118984)). As we anticipate a strong co-evolution among the corresponding genes, we used these sets as positive controls for co-evolution in general. Finally, we subsampled the genomes of organisms that were not included in the proteome database for 30 random loci containing 8 to 10 genes (S3 Table; sequences available at the GitHub repository https://github.com/gvignolle/FunOrder (doi:10.5281/zenodo.5118984)). We termed this control set „sequential GCs“. This set should represent the random degree of co-evolution based only on genomic vicinity. Notably, due to the randomness of the sampling, the sequential GCs may also contain evolutionary linked genes.

### Calculation of MEM and determination of thresholds for co-evolution

As the thresholds for the strict and/or evolutionary distance for the analysis of protein co-evolution are database dependent, we needed to define these thresholds manually. To this end, we performed a manual comparison of the phylogenetic trees of genes anticipated to be co-evolved and of not presumably co-evolved genes. As positive control datasets (anticipated co-evolution), we used the essential genes within the positive control BGCs. As negative control data set (anticipated to not have co-evolved), we used the genes in the random GCs. For the manual tree comparisons, we considered the topology (defined in S4 Table), branch lengths, number of nodes, and shared leaves of the trees and calculated the manual evaluation measure (MEM) according to the definitions in S5 Table. We calculated the MEM for each gene tree pair of the positive and the negative control data sets (S6 and S7 Tables, respectively). The measure ranges from 3 (same) to 0 (no shared leaves). The MEM values of each pair-wise tree comparison were then manually reconciled with the corresponding strict and the combined distance measures obtained from the treeKO analysis and the subsequent R script, respectively. The procedure is exemplary described for the 2-Pyridon-Desmethylbassianin (dmb) BGC from *Beauveria bassiana* in S1 File. Based on these manual comparisons, we defined the threshold values for strict and combined distances in the following: two genes are considered as co-evolved if the strict distance value is less than 0.7 or if the combined distance is equal to or less than 60 percent of the maximum value in the combined distance matrix of the analysed set.

### Calculation of the Internal co-evolutionary quotient (ICQ)

The internal co-evolutionary quotient (ICQ) expresses how many genes in a GC or proteins in a protein set are co-evolved according to the previously defined threshold for strict and combined distances within the distance matrices of an analysed GC (or protein set). To calculate the ICQ, each protein is compared with every other protein. The total number of all possible pairwise comparisons is 2* [d*(d-1)] for d proteins. The ICQ was calculated using Eq 1, resulting in values between 0 and 1, with 1 representing no co-evolved genes, and 0 representing that most genes are co-evolved with each other in the insert GC.


$$ICQ=1-\left\{\frac{g}{2*[d*(d-1)]}\right\}$$ Eq 1

ICQ = internal co-evolutionary quotient; g = number of strict distances < 0.7 and combined distances < = (0.6 * max value of the combined distance matrix) in all matrices (visualized in the heatmaps); d = number of genes in the GC.

### Manual interpretation of the FunOrder output

The FunOrder outputs three different visualizations (heatmap, dendrogram, PCA) each of the strict and combined distance matrices among the genes (or proteins) of an inserted GC (or protein set). These visualizations need to be interpreted manually. For the manual interpretation, we first searched for genes that clustered together with the core enzyme(s) in any of the three visualisations of the strict distance. The definition of the clusters needs to be performed carefully keeping the biological background (gene predictions) in mind. For instance, a cluster containing typical tailoring enzymes (e.g., hydrolases, P450 cytochrome oxidases, FAD-containing enzymes, etc.) and/or further essential genes (e.g., transcription factors or transporters) make sense, whereas clusters containing a lot of genes encoding for unknown genes and/or genes that are unlikely to be involved in the biosynthesis of a secondary metabolite) do not make sense. Next, clustering in the visualizations of the combined distances is considered. As the combined distance also contains information about the speciation history, it may be used to add further genes to the list of “detected genes”. Notably, this needs to be critically evaluated and decided on a case-to-case basis, taking the gene predictions into account. Please also refer to S2 File for a detailed step-by-step description of the interpretation procedure, the exemplary analysis of the lovastatin BGC from *A*. *terreus* in the results, and S3 File and S4 File for the exemplary analysis of two unknown BGCs.

### Performance evaluation

To test the robustness of FunOrder, we analysed 42 completely randomly generated synthetic GCs. To test whether the FunOrder method can be used to detect co-evolution within GCs (or protein sets), we calculated the ICQ for different control sets and compared the results in a kernel density plot. To evaluate the performance of the FunOrder method regarding its capability to identify presumably co-evolved essential genes (as defined in S1 Table) and to distinguish them from (presumably not co-evolved) gap genes and genes outside of the BGC via the detection of co-evolution, we performed a manual interpretation of 30 empirically characterized BGCs (Table 2) as described above. Genes that clustered together with the core enzyme(s) according to the procedure described above were considered as „detected“. Then we counted the total number of (1a) detected essential genes or (1b) detected biosynthetic genes, (2a) not detected essential genes or (2b) not detected biosynthetic genes, (3) detected gap and extra genes, and (4) not detected gap or extra genes in all BGCs, and defined (1a or 1b) as true positives (TP), (2a or 2b) as false negatives (FN), (3) as false positives (FP), and (4) as true negatives (TN). The values were used for a final statistical evaluation of FunOrder as suggested by Chicco and Jurman [198].

## Results and discussion

### Applicability of FunOrder for the detection of co-evolution

First, we analyzed the 42 synthetic negative control GCs with the FunOrder software. We could not find any sequence similarities with the empirically optimized fungal proteome database, demonstrating the robustness of the FunOrder method towards non-biological random amino acid sequences. Consequently, the 42 synthetic negative control GCs were not considered in the following.

Next, we performed FunOrder analyses of different control GCs and protein sets and calculated the internal co-evolutionary quotients (ICQs) using Eq 1. The ICQ is a value for the relative amount of co-evolutionary relations among the genes (or proteins) in a given GC or protein set. An ICQ of 0 means that most genes (or proteins) are co-evolved with each other. An ICQ of 1 means, that no co-evolution can be detected using the defined thresholds. As negative control for co-evolution, we used 60 randomly assembled negative control GCs (random GCs, S8 Table). The random GCs were compiled by subsampling different proteomes, to minimize the chance of random, unwanted co-evolution in the clusters. As positive control for co-evolution we used 10 protein sets from conserved metabolic pathways of different ascomycetes (S2 Table), termed „Biosynthetic pathways“, or „BioPath“. Given, that the proteins are part of the conserved primary metabolism and that their enzymatic functions are interrelated, we can assume a high level of internal co-evolution among the proteins within these protein sets. As control for the basic co-evolutionary value of co-localized (or sequential) genes, we used 30 random genetic loci containing 8 to 10 genes (S3 Table). We termed this control set „sequential GCs“. As test set for BGCs of the secondary metabolism in ascomycetes we used 30 empirically characterized BGCs (Table 2, S1 Table), also termed positive control BGCs.

**Fig 3:**
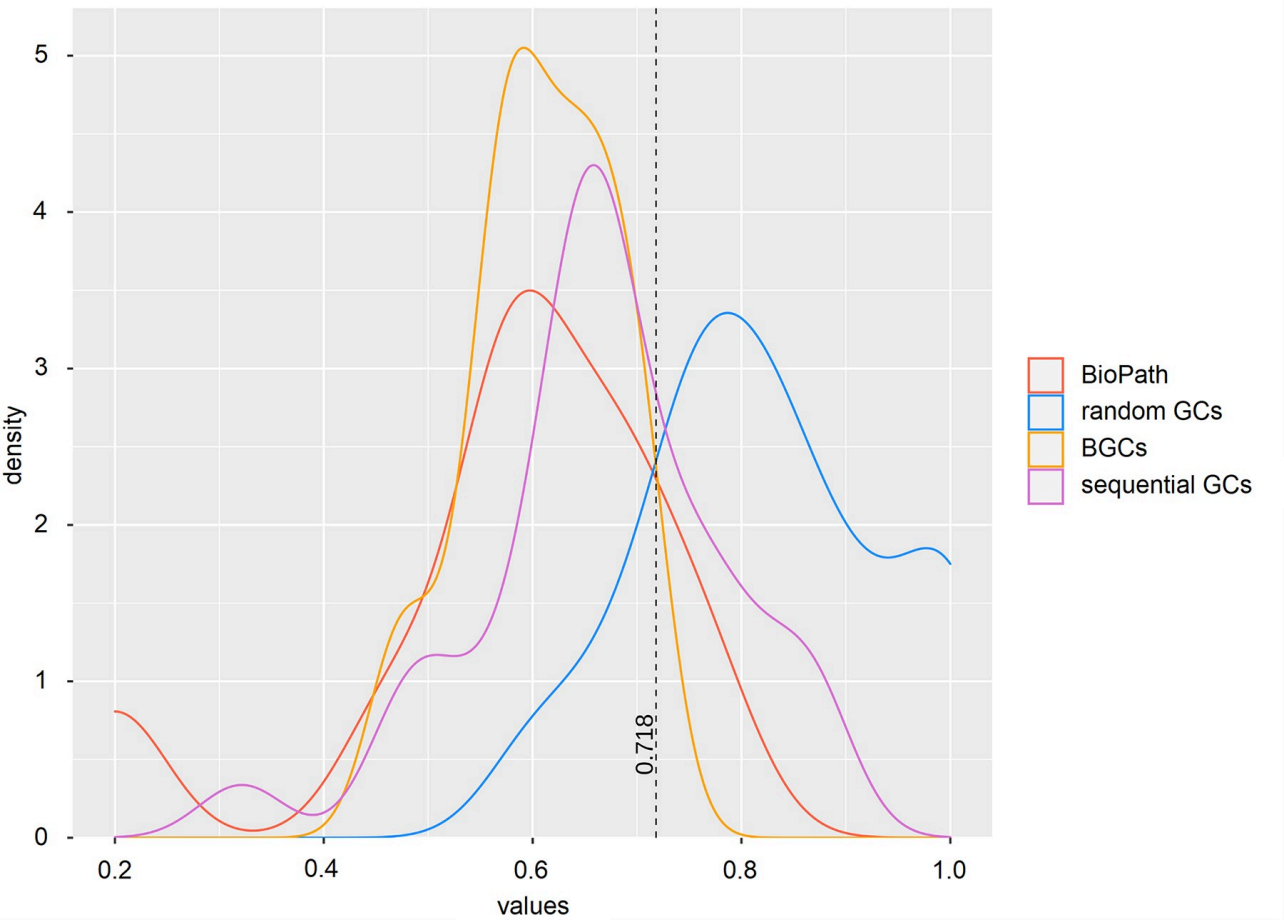
Kernel density plot of the ICQ values for co-evolutionary linked enzymes of different control sets. BioPath, protein sets of conserved biosynthetic pathways of the primary metabolism (S2 Table); random GCs, randomly assembled protein sets from 134 fungal proteomes (Table 1); BGCs, previously empirically characterized fungal BGCs (Table 2); sequential GCs, co-localized genes from random loci of different ascomycetes (S3 Table).

We compared the ICQs of the different sets in an ANOVA (S5 File) and in a kernel density plot (Fig 3). We found that the ICQs for the random GCs were significantly different from all the other sets, demonstrating that the workflow of the FunOrder method can be used to detect co-evolution, that the ICQ is a meaningful measure to represent the content of co-evolutionary relationships within a GC or protein set, and that the manually defined thresholds for strict and combined distances are applicable to define co-evolution within GC or proteins sets. Based on these results, we defined the threshold of the ICQ for biologically relevant co-evolution within a GC as the point of intersect between the random GCs and the BGCs (0.718). GCs with an ICQ above this threshold do not contain significantly more co-evolutionary connections among the contained genes than randomly assembled GCs.

To our surprise, we could not detect a statistically significant difference between the sequential GCs and the positive control GCs. However, the maxima for the BioPath proteins and the BGC are at the same value and the shape of the corresponding density plot is remarkably similar (Fig 3), whereas the maximum of the sequential GC is shifted towards the random GCs and the shape of the curve is different to the two positive control sets (Fig 3). These results indicate, that using only the absolute values of strict and combined distance may not be enough to distinguish co-evolutionary linked genes within the context of co-localized genes, but that the distances need to be assessed and interpreted in a case-by-case scenario considering the biological background and context of the analyzed GC.

### Exemplary analysis of the lovastatin BGC (lov)

**Fig 4:**
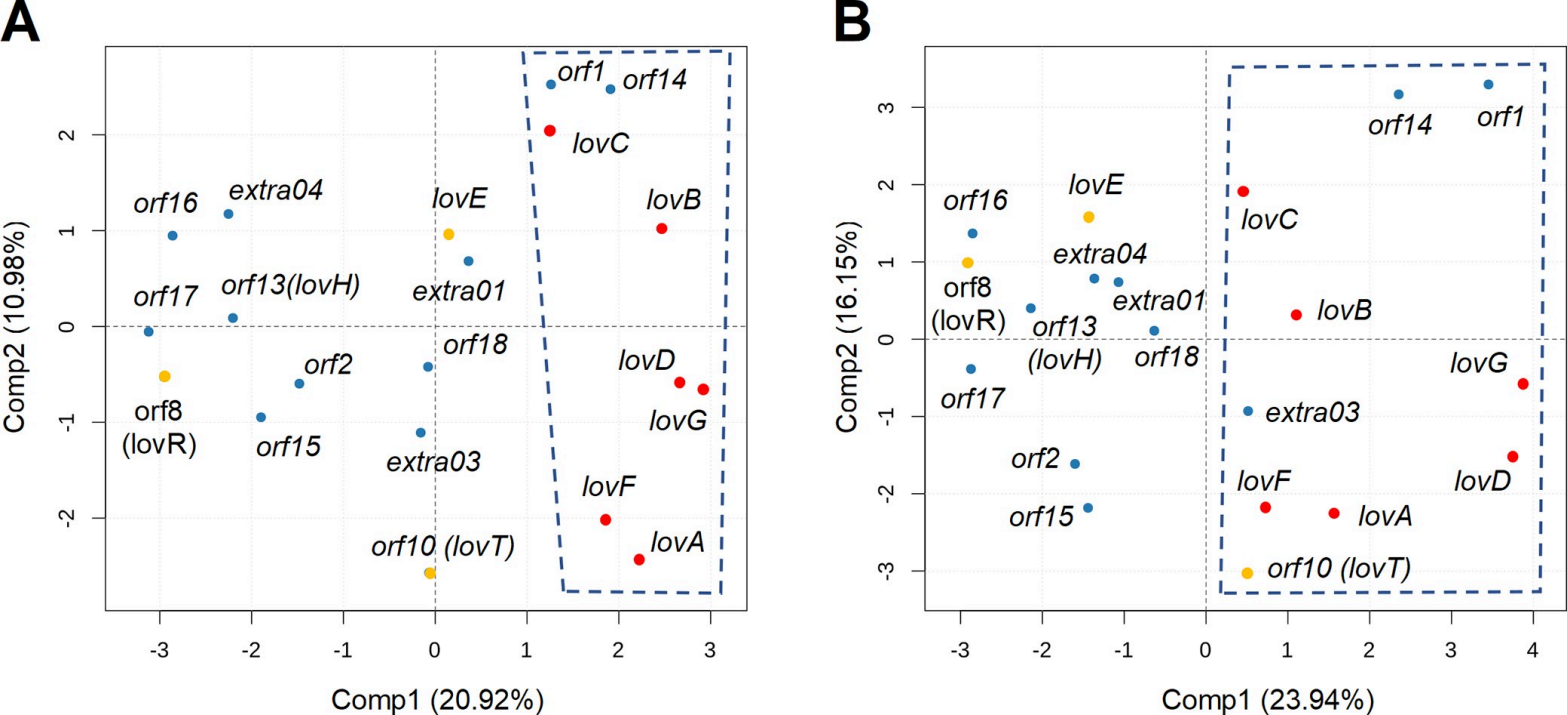
A selection of the standard output of the FunOrder analysis of the lovastatin BGC (lov). Score plots of the first two principal components from a PCA performed on the strict distance matrix (A) and on the combined distance matrix (B). The biosynthetic genes and the further essential genes are indicated in red and gold, respectively. Clusters in the PCA are indicated by the dashed boxes.

The FunOrder method allows the detection of co-evolved genes within a set of genes or proteins. As mentioned, we speculate that essential genes in BGCs are co-evolving and can therefore be differentiated from gap genes. In this context, the application of FunOrder might be used to detect the essential or at least the biosynthetic genes in BGCs. The software package of the FunOrder method calculates two distance matrices for the proteins within an input GC representing the evolutionary similarities (based on pair-wise comparisons of the phylogenetic trees using the treeKO tool [45]). First, we tried to use the previously defined thresholds for the strict and combined distances to automatically detect the co-evolutionary relations in BGCs. As insinuated above, this proofed not to be a successful strategy (not shown). We speculate, that the evolutionary similarities or distances among neighbouring genes are highly location specific and that the absolute values are therefore not meaningful as general thresholds. However, as the underlying strategy and method is clearly able to detect co-evolution (Fig 3), we speculated that the obtained data may need to be represented in different forms and/or reduced. Consequently, we added the following data visualizations to the FunOrder pipeline. The strict and combined distances are visualized in a heatmap and clustered by higher similarities (complete linkage method). Next, the Euclidean distances within the scaled distance matrices are calculated and clustered (hierarchical clustering) using the Wards minimum variance method aiming at finding compact spherical clusters, with the implemented squaring of dissimilarities before cluster updating. The clustering is visualized in dendrograms. Finally, the principal components of the data are represented in a score plot. Here, we exemplary describe the manual interpretation of these visualizations (S6 File and Fig 4) with the aim to detect co-evolution within the lovastatin BGC of *A*. *terreus* (lov, Fig 1). Please refer also to the step-by-step description on how to interpret the FunOrder output in S2 File.

For the analysis of the lovastatin BGC, we first had a look at the heatmap representing the strict distance matrix (S6 File). Therein all biosynthetic genes (*lovA-D*, *F*, *G*; Fig 1, red arrows) are clustering together with each other and with the gap gene *orf1*, although not all inter-gene distances were below the previously defined threshold (S6 File, heatmaps). This demonstrates again that, evaluating only the numerical values (regardless of the concrete thresholds) is not enough for a thorough analysis of a BGC. It is necessary to consider the distances within the genomic context by comparing all provided visualisations. The biosynthetic genes of lovastatin (*lovA-D*, *F*, *G*) also formed distinct clusters in the dendrograms and in the PCA of the strict distance (S6 File and Fig 4A) In our experience, it was often helpful to additionally take the combined distance values into consideration to get a more comprehensive picture of the BGC. As mentioned before, the combined distance also considers speciation history. In the case of the lovastatin BGC, *orf10* and *extra03* clustered together with *lovA*, *B*, *D*, *F*, *G* in the PCA of the combined distance (Fig 4B). The gene *orf10* encodes for an MFS (major facilitator superfamily) transporter, which warrants adding it to the „detected genes“; the transporter is actually necessary for the export of lovastatin [17] (Fig 1). The gene *extra03* is predicted to encode for an alpha-glucuronidase (AguA) which is involved in the hydrolysis of xylan. Therefore, the clustering only in combined distance matrix does not justify classifying the gene *extra03* as „detected“. The other two „further essential genes“, *lovE and orf8* did not cluster together with the biosynthetic genes in any visualizations of the distance matrices (Fig 4 and S6 File)). LovE is a transcription factor and the main regulator of the lovastatin cluster [17] and essential for the lovastatin biosynthesis in the native organism, although it is not directly part of the biosynthetic pathway. The gene *orf8* encodes for a 3-hydroxy-3-methylglutaryl coenzyme-A (HMG-CoA) reductase, which is the target of statins [199] and in this case is conveying self-resistance to lovastatin [200]. These results suggest that these two genes did not undergo the same evolutionary process as the biosynthetic genes. This is in accordance with the „brick and mortar”model suggested by Medema et al. [40]. The biosynthetic genes represent a co-evolving „brick“, that is integrated into the biological context of *A*. *terreus* via the „mortar”that are the further essential genes.

This exemplary analysis demonstrates how the different data output formats of the software package need to be considered and compared manually, to decide on which genes are co-evolutionary linked and likely to be involved in the biosynthesis of a secondary metabolite. When considering only one output, one might get a distorted view of the analysed BGC. Notably, we did not intend to leave this step up to automation, because the human (expert or child) pattern recognition and mind still outperforms artificial intelligence (AI) algorithms and machine learning algorithms in this regard [201]. Please also refer to S3 File and S4 File in which we describe the analysis of two yet undescribed BGCs.

### Speed and scalability of the software

As the empirically optimized proteome database contained only 134 fungal proteomes, we were able to use the blastp algorithm for sequence similarity search. The analysis of the lovastatin BGC of *A*. *terreus* (lov) with 17 genes, took 1 h 19 m 48 sec real time using 22 threads on an Ubuntu Linux system with 128 GB DDR4 RAM. The same analysis took 6 h 54 m 50 sec real time using 3 threads and 5 h 48 m 50 sec using 4 threads on a Linux Mint Laptop, demonstrating that the analysis of such a large cluster as the lovastatin cluster is fast and feasible. The number of threads can be defined, to increase the scalability and the overall performance.

### Performance evaluation

**Fig 5:**
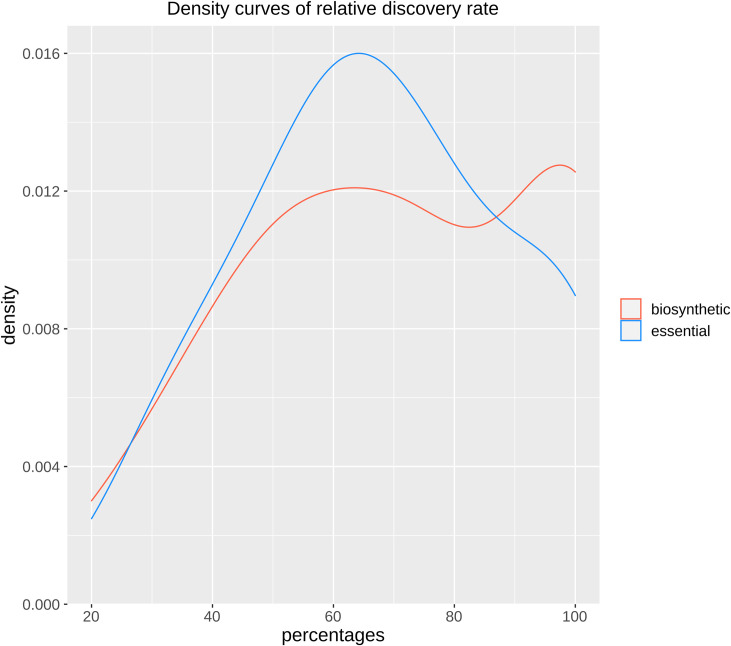
Kernel density plots of the relative discovery rate of essential or biosynthetic genes in 30 tested fungal BGCs.

Up to this point, we demonstrated that the FunOrder method can be used to detect the overall level of internal co-evolutionary relations within a GC or set of proteins. We demonstrated that similar levels of co-evolutionary relations occur among the genes in BGCs and among proteins of conserved metabolic pathways of the primary metabolism, and that these positive control sets can be distinguished from negative control GC, containing randomly stringed together proteins from different organisms with a threshold of 0.718 for the ICQ (Fig 3). Further, we showed that the values of strict and combined distances need to be visualized in different forms and then interpreted manually to detect co-evolution of individual genes within fungal BGCs. Next, we aimed to test, whether the detection of co-evolved genes is indeed a useful approach to identify the essential genes in fungal BGCs. To this end, we analysed the 30 empirically verified BGCs (Table 2) as described for the lovastatin cluster before. We looked for genes that are co-evolutionary linked with the core biosynthetic gene. These genes were considered as “detected”. The “detected” genes sets were compared to the previously empirically obtained set of essential genes and classified the genes in true positives (TP), false negatives (FN), false positives (FP), or true negatives (TN) (S1 Table). To test and evaluate, how well FunOrder is performing in detecting either all essential or just the biosynthetic genes, we determined two different sets of TP and FN. TPs were either all detected essential genes, or all detected biosynthetic genes. Accordingly, FNs were either all not detected essential genes or all not detected biosynthetic genes (S1 Table). In both cases, FPs were all detected gap and extra genes, and TNs were all not detected gap and extra genes (S1 Table) because it makes biologically no sense to define a „detected”further essential gene as a FP, even when defining detected biosynthetic genes as TP. For an initial performance estimation, we calculated the percentages of detected essential and biosynthetic genes (S1 Table) and compiled them in a kernel density plot (Fig 5). More than 75% of all essential genes and biosynthetic genes were found to be co-evolving using the FunOrder method in 13 and 16 BGCs (out of 30 BGCs), respectively. The curves in the density plot also differ at high percentages; nearly all (above 90%) biosynthetic genes could be detect in more cases than nearly all essential genes. These two observations point in the direction, that especially the biosynthetic genes share a more coherent co-evolutionary history and can thus be identified by looking for co-evolved genes in BGCs. Obviously, not all essential genes in all BGCs are co-evolving and/or can be detected as co-evolved with this method. This is at least partly based on the biological background. Each BGC has a unique evolutionary background and needs to be interpreted individually. The FunOrder method offers additional information about co-evolution for already defined BGCs and may be useful in deciding which genes might be most relevant when studying a BGC.

For a stringent statistical evaluation, we calculated the normalized Matthews correlation coefficient (normMCC) and other classical metrics and global metrics (Table 3) as indicated by Chicco and Jurman [198] based on the previously defined TP, FN, FP, and TN (S1 Table). To determine the degree of balance between positive and negative controls we calculated the no-information error rate ni which is best for balanced test sets with the value 0.5. The obtained values of 0.5084 and 0.5444 allowed for the usage and confirmed the validity of the classical metrics such as F1 score and Accuracy. The FunOrder method displays overall high metrics in identifying essential and/or biosynthetic genes in a BGC. Despite the differences between biosynthetic and essential genes in Fig 5, we could not detect strong differences in the overall statistical assessment. FunOrder can be used to detect essential and biosynthetic genes in a BGC based on protein family co-evolution with a accuracy of 0.7215 and 0.743, respectively.

**Table 3:** Statistical evaluation of the performance of FunOrder in detecting relevant genes in BGCs.

	essential genes	biosynthetic genes
Sensitivity	0.6349	0.6615
Specificity	0.8112	0.8112
Precision	0.7766	0.7457
Negative Predictive Value	0.6823	0.7412
False Positive Rate	0.1888	0.1888
False Discovery Rate	0.2234	0.2543
False Negative Rate	0.3651	0.3385
Accuracy	0.7215	0.743
F1 Score	0.6986	0.7011
Matthews Correlation Coefficient	0.4524	0.4797
Normalized Matthews Correlation Coefficient	0.7262	0.73985
No-information error rate ni	0.5084	0.5444

### Concluding remarks

The FunOrder method was created to identify the essential genes in a BGC and distinguish them from gap genes based on the hypothesis that the essential genes are co-evolutionary linked. We evaluated this method and simultaneously tested the underlying hypothesis using different control sets of genes and proteins, respectively. We observed on the one hand that co-evolutionary linkage in fungal BGCs is commonly occurring—especially within the biosynthetic genes, and on the other hand that the FunOrder method can be used to detect the biosynthetic genes within BGCs and to some extent also the further essential genes. We would like to stress that this method is delivering data on co-evolution, that needs to be critically evaluated and interpreted keeping the biological background in mind, and that FunOrder is not to be considered a stand-alone tool but meant to deliver supplementary data about co-evolution within predefined BGCs.

During the testing and evaluation, we encountered several cases of ambiguous results, where the different visualizations clustered different genes together. One way to handle such ambiguous results is to critically assess the results by considering the gene predictions. We further suggest adding and/or removing genes at the edges of the BGC and re-running the analysis. This might change the clustering behaviour and clarify the results. Alternatively, homologous BGCs from other fungi may be analysed by FunOrder and the clustering of the corresponding genes compared to the initial BGC.

The basis but also limitation for the method is the database [42]. Here we used a specific set of proteomes (Table 1) and were thus able to detect co-evolved genes in ascomycetes. Notably, the underlying strategy and workflow of FunOrder can be adapted to analysing genomic regions in other phyla, orders, or even kingdoms by using different databases. In case a larger database is integrated into the software package, alternative search algorithms, such as DIAMOND [202] or HMMER (similarity search using hidden Markov models) [203] might be used instead of blastp to enhance the performance. Nevertheless, each novel database, even if only one single proteome would be introduced in an existing database, will have to be verified and validated.

In this study, we looked for genes that share the same or a similar evolutionary background with the core genes of BGCs and could demonstrate that FunOrder is a fast and powerful method that can support scientists to decide which genes of a BGC are promising study objects. Notably, the application of this method is not limited to fungal BGC. It can be used for any applications where information of a shared co-evolution can contribute to a better understanding. FunOrder with the existing ascomycete database might already be used for a genome wide analysis of co-evolving transcription factors or detection of functionally connected protein-protein interactions [42]. As a future perspective, FunOrder might be even used for the analysis of total proteomes to detect evolutionary linked genes.

## Supporting information

S1 Table Empirically tested BGCs used as control set in this study. (XLSX)

S2 Table Protein sets of conserved metabolic pathways of the primary metabolism. (XLSX)

S3 Table Sequential GCs used in this study. (XLS)

S4 Table Definition of topology. (PDF)

S5 Table Parameters used to calculate the manual evaluation measure (MEM). (PDF)

S6 Table Calculation of MEM values for positive control BGCs. (XLSX)

S7 Table Calculation of MEM values for negative control GCs. (XLSX)

S8 Table Random GCs used in this study. (XLSX)

S1 File Exemplary MEM analysis of the dmb BGC. (PDF)

S2 File Step-by-step explanation for the manual interpretation of the FunOrder output. (PDF)

S3 File Exemplary interpretation of the FunOrder output of an unknown fungal BGC 1. (PDF)

S4 File Exemplary interpretation of the FunOrder output of an unknown fungal BGC 2. (PDF)

S5 File ANOVA for the ICQ values of the control and tests GCs and protein sets, respectively. (PDF)

S6 File FunOrder output of the Lovastatin BGC from *A*. *terreus* (lov). (PDF)
