# Supplementary material for: FunOrder: A robust and semi-automated method for the identification of essential biosynthetic genes through computational molecular co-evolution
Source: PLoS Comput Biol. 2021 Sep 27;17(9):e1009372. doi: 10.1371/journal.pcbi.1009372 (PMC8476034; doi:10.1371/journal.pcbi.1009372)
Supplement: S4 Table — (PDF) [file pcbi.1009372.s004.pdf]

S1 Table. Definition of topology.

Topology	Definition
**same**	min. 8 similar species, same topology with only little exceptions, colour 70-100%
**very similar**	min.5 similar species, similar topology, colour min. 70%
**similar**	distance < 2, colour min. 50%
**somewhat similar**	either 1 or 2 similar species with distances < 0.5 and nodes <3, or more species but only little similarities
**different**	no similarities or only 1 similar species
