# Supplementary material for: FunOrder: A robust and semi-automated method for the identification of essential biosynthetic genes through computational molecular co-evolution
Source: PLoS Comput Biol. 2021 Sep 27;17(9):e1009372. doi: 10.1371/journal.pcbi.1009372 (PMC8476034; doi:10.1371/journal.pcbi.1009372)
Supplement: S5 Table — (PDF) [file pcbi.1009372.s005.pdf]

S2 Table. Parameters used to calculate the manual evaluation measure (MEM).

ΔBranch lenghth	ΔNodes	Color	Topology	MEM
**0 – 0.5**	0	blue	same	3
**0.5 - 1**	1	-	very similar	2.5
**1 – 1.5**	2	green	Similar	2
**1.5 - 2**	3	-	somewhat similar	1.5
**> 2**	> 4	yellow	different	1
