# Supplementary material for: FunOrder: A robust and semi-automated method for the identification of essential biosynthetic genes through computational molecular co-evolution
Source: PLoS Comput Biol. 2021 Sep 27;17(9):e1009372. doi: 10.1371/journal.pcbi.1009372 (PMC8476034; doi:10.1371/journal.pcbi.1009372)
Supplement: S1 File — (PDF) [file pcbi.1009372.s009.pdf]

Representative calculation of the manual evaluation measure (MEM) and comparison of the results to the FunOrder output for determination of thresholds based on the 2-Pyridon-Desmethylbassianin (dmb) BGC from *Beauveria bassiana*

Two phylogenetic trees, each representing a gene within a cluster in the context of our empirically optimized database, were compared. For each tree comparison we first determined if there were similar leaves (similar Species) between the two trees. If yes, branch length differences, node-differences, branch colours and overall topology between the leaves and the query were determined.
The branch lengths were measured, and the differences then calculated. The nodes between a species and the query were counted and compared to the number of nodes of the other phylogenetic tree. The branch colour describes the similarity to the most common node based on the Robinson-Foulds (RF) distance. 0 to 40% similarity was defined as “yellow”, 40 – 66,6% similarity was defined as “green” and the rest was defined as “blue”. For each of the four measures average pairwise distances were determined. If the trees contained more than two similar leaves, another average would be calculated of the resulted average measures, called manual evaluation measure (MEM). These MEMs (the higher the MEM the higher the similarity) (S6 Table) were put together in matrices to calculate heatmaps, dendrograms and PCA to evaluate the FunOrder output based on the treeKO algorithm (lower distances indicated higher similarities).

Table 1 FunOrder strict matrix

	dmbS	*dmbA*	*dmbB*	*dmbC*
dmbS	0	0.524	0.864	0.672
dmbA	0.524	0	0.793	0.533
dmbB	0.864	0.793	0	0.807
dmbC	0.672	0.533	0.807	0

Table 2 FunOrder evol matrix

	dmbS	*dmbA*	*dmbB*	*dmbC*
dmbS	0	0.12	0	0.343
dmbA	0.153	0	0	0
dmbB	0	0	0	0.103
dmbC	0.322	0	0.103	0

The 2-Pyridon-Desmethylbassianin (dmb) BGC from *Beauveria bassiana* (BGC0001136) consists of 4 genes (*dmbA*, *dmbB*, *dmbC*, *dmbS*). According to literature *dmbS* and *dmbC* are needed for the production of 2-Pyridon-Desmethylbassianin (Heneghan, Yakasai et al. 2011).

We can now compare the highest MEMs to the corresponding strict distances from the FunOrder output to determine if they are comparable. The highest MEM was calculated for the *dmbA:dmbC* comparison (MEM =2.61), in the FunOrder output for the strict distance this comparison (0.533) is next to lowest (Table 1 and S6 Table). The evolutionary distance for this comparison is 0 (Table 2). Next the the *dmbA:dmbS* comparison (MEM = 2.53) is the lowest in the strict matrix but has the evolutionary distance (0.12 and 0.153) (The differences between the two values are created due to the treeKO algorithm and the decision which tree to use as reference). This clarifies the strength of the MEMs, that they consider evolutionary history in one value, and the introduction of the combined distance measure, where speciation is considered with the strict distance as background. The comparison *dmbS:dmbC* had a MEM of 2.4 and a strict distance of 0.672. When comparing the clustering of the Ward`s minimum variance on the unscaled data, we can further observe similar clustering (Figure 1 A and B).

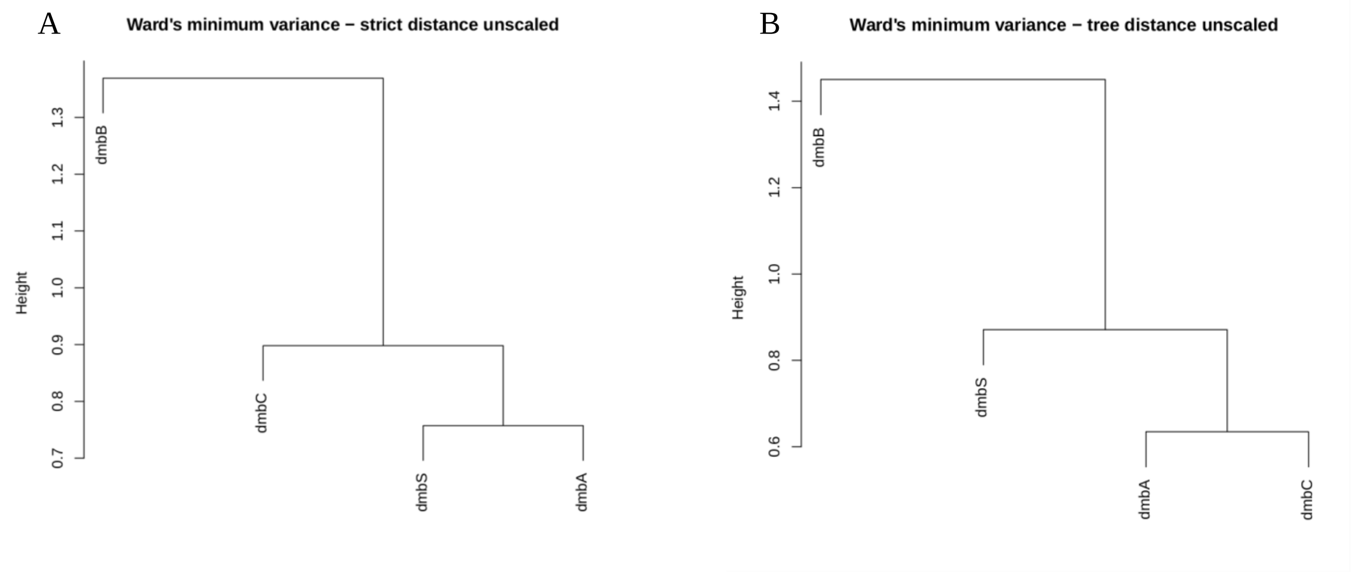

Figure 1 A – Standard output of the FunOrder analysis of the 2-Pyridon-Desmethylbassianin BGC of *Beauveria bassiana* (BGC0001136) (dmb). Dendrogram based on the Euclidean distance within the unscaled strict distance matrix clustered using Ward's minimum variance method aiming at finding compact spherical clusters, with the implemented squaring of the dissimilarities before cluster updating. **B** – Dendrogram based on the Euclidean distance within the unscaled MEM matrix of the 2-Pyridon-Desmethylbassianin BGC of *Beauveria bassiana* (BGC0001136) (dmb) clustered using Ward's minimum variance method aiming at finding compact spherical clusters, with the implemented squaring of the dissimilarities before cluster updating.

In this example, we can see clear similar clustering (Figure 1) between the dendrograms based on the MEM values and those based on the strict distances. Further, we can see how strict distance values below 0.7 reflect manual determination and refinement of co-evolution (Table 1, Table 2 and S6 Table). We can further see how the manual determination of co-evolution takes speciation history into account and therefore the validity of the introduction of the combined distance, which resembles the manual comparison. We further compared Heatmaps and PCAs and performed these comparisons for all analysed positive control BGCs and negative control gene clusters.

References:

Heneghan, M. N., A. A. Yakasai, K. Williams, K. A. Kadir, Z. Wasil, W. Bakeer, K. M. Fisch, A. M. Bailey, T. J. Simpson, R. J. Cox and C. M. Lazarus (2011). "The programming role of trans-acting enoyl reductases during the biosynthesis of highly reduced fungal polyketides." Chemical Science **2**(5).
