# Supplementary material for: FunOrder: A robust and semi-automated method for the identification of essential biosynthetic genes through computational molecular co-evolution
Source: PLoS Comput Biol. 2021 Sep 27;17(9):e1009372. doi: 10.1371/journal.pcbi.1009372 (PMC8476034; doi:10.1371/journal.pcbi.1009372)
Supplement: S2 File — (PDF) [file pcbi.1009372.s010.pdf]

Standard Operation Procedure for the Interpretation of the FunOrder Results

1) The internal co-evolution quotient (ICQ) gives information about the total co-evolution within an insert BGC. If the ICQ is above 0.718, the content of the co-evolution is not significantly different to randomly assembled GCs. Such BGCs must be interpreted with extreme caution. It might be worth trying to add or remove some gene from the edges of the BGC and re-run the FunOrder analysis.
2) The heatmap based on the strict distances is representing the calculated raw data (treeKO output) and can be used for an initial, general overview. Genes with a shared co-evolution may cluster together and form distinct clusters in the heatmap (regardless of the absolute values) and the corresponding dendogram. Such clusters may be a good first indication for co-evolution but are not a necessity.
3) Next, the dendrogram based on the euclidean distances within the scaled strict distance matrix is inspected. In the context of BGCs, it is sensible to look for genes that cluster together with the core enzyme(s) in the dendrogram. Notably, the dendrogram is still a representation of the complete data set. Clustering (or the absence of clustering) may not only be caused by co-evolution but also by potential generated noise.
4) Therefore, the final and crucial step to detect co-evolving genes is to consider the PCA-plot of the strict distance. First, the percentage described by the principal components must be compared and taken into account for the clustering. For example, if PC1 (x-axis) describes 50% of the data and PC2 (y-axis) describes 10% of the data, longer vertical distances between genes are allowed, because of the stronger horizontal impact. Genes that cluster together with the core enzyme(s) are highly likely to share a similar co-evolution.
5) Genes that are clustering together with the core enzyme(s) in any of the three visualisations are considered ‘detected’ and can be anticipated to share a similar co-evolution.
6) Finally, the steps 2 – 4 are repeated with the visualizations of the combined distances. This may add further genes to the pool of ‘detected’ genes.
