# Supplementary material for: FunOrder: A robust and semi-automated method for the identification of essential biosynthetic genes through computational molecular co-evolution
Source: PLoS Comput Biol. 2021 Sep 27;17(9):e1009372. doi: 10.1371/journal.pcbi.1009372 (PMC8476034; doi:10.1371/journal.pcbi.1009372)
Supplement: S3 File — (PDF) [file pcbi.1009372.s011.pdf]

Phialocephala scopiformis biosynthetic gene cluster analysis with FunOrder

To give an example for the FunOrder analysis of an undescribed biosynthetic gene cluster (BGC), we chose a putative Type I polyketide synthase (T1pks) BGC from the fungal conifer needle endophyte *Phialocephala scopiformis* (1) (located on scaffold NW_017263581, 125525-172708 nt). This cluster was predicted with antiSMASH 4.3.0 (2) (Figure 1) and the output was directly analyzed with FunOrder.

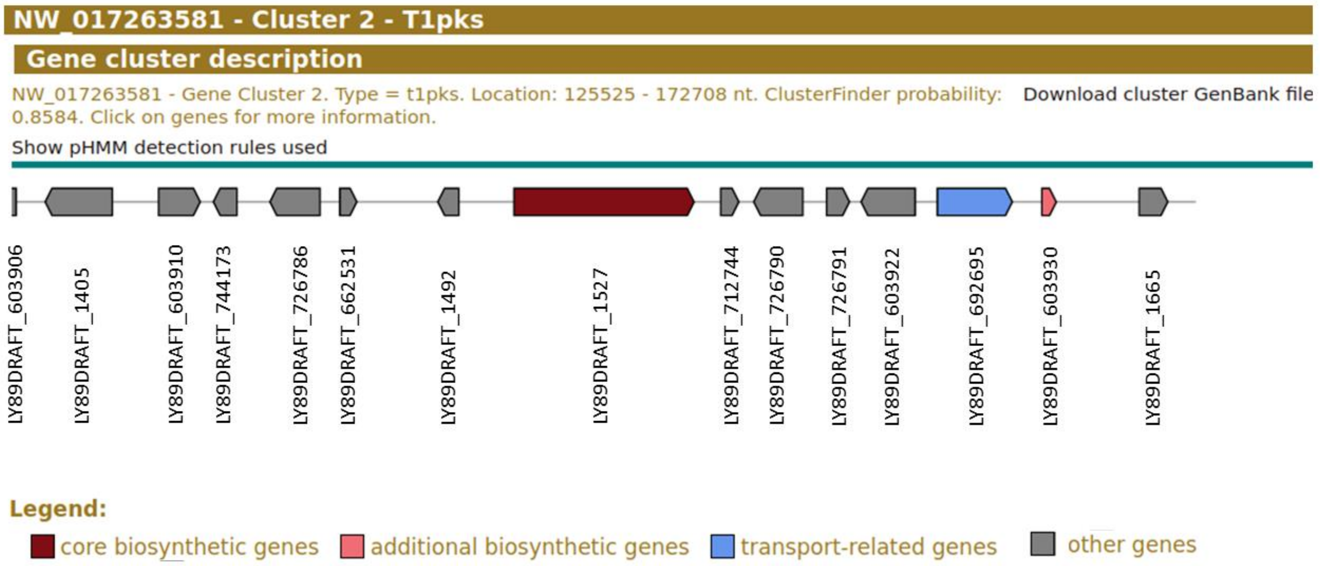

Figure 1 Screenshot of the cluster defined by antiSMASH.

The first step of the analysis was to inspect the internal co-evolutionary quotient (ICQ) calculated for this specific cluster. The ICQ was 0.5727, which is below the previously defined threshold for relevant co-evolution detected of 0.718. We therefore continued with the inspection of the heatmap based on the strict distance matrix (Figure 2). The color key in the heatmap is a direct visualization of the values of the strict distance, they are clustered based on a calculated dendrogram based on the complete linkage method. We observed a first indication of which genes might share a potential co-evolution with the core enzyme LY89DRAFT_1527 (marked as LY89DRAFT_1527_T1PKS in all figures). The inspection of figure 2 indicated LY89DRAFT_1492 (annotated as hypothetical protein and after a sequence similarity search with blastp (3) against the non redundant protein database revealed as putative serine hydrolase), LY89DRAFT_603930 (annotated as NAD(P)-binding protein and a smCOG short chain dehydrogenase/reductase) and LY89DRAFT_603910 (annotated as type 1 phosphodiesterase/nucleotide pyrophosphatase) as sharing relatively lower distance values among each other.

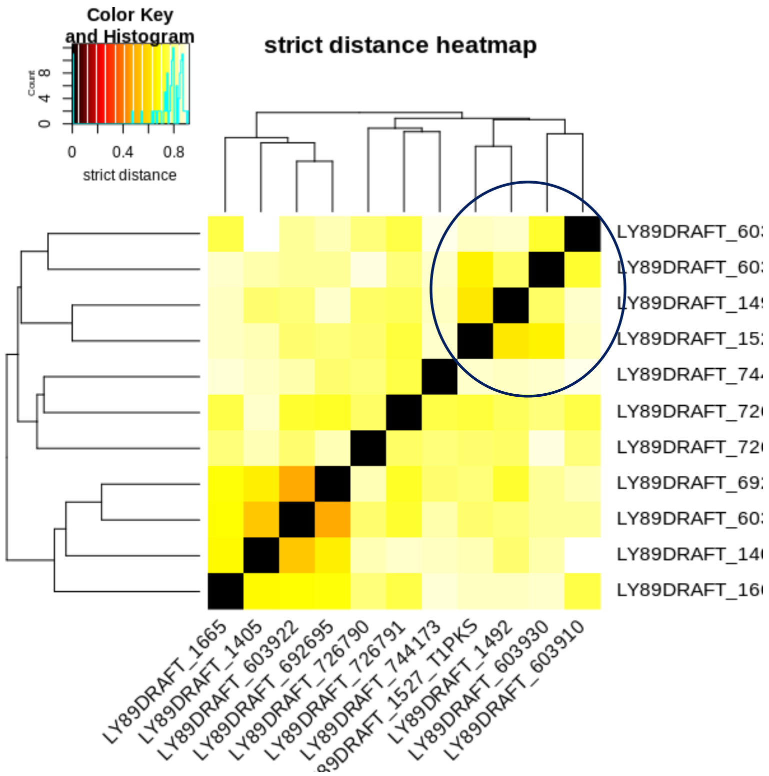

Figure 2 Standard output of the analysis of the putative T1pks BGC of *Phialocephala scopiformis* (located on scaffold NW_017263581, 125525-172708 nt). Heatmap of the strict distance matrix. The clustering mentioned in the text is indicated by a blue circle.

Next we examined the dendrogram (Figure 3) based on the Euclidean distances within the scaled strict distance matrix clustered using Ward´s minimum variance method aiming at finding compact spherical clusters, with the implemented squaring of the dissimilarities before cluster updating. Again, we looked for the core enzyme LY89DRAFT_1527. This enabled us to determine that LY89DRAFT_1492 seems to share the strongest similarity in strict distances with LY89DRAFT_1527. Clustering relatively close to the T1pks were LY89DRAFT_603930 and LY89DRAFT_603910. This clustering considered the complete strict

distance matrix, including potential noise, which could distort the detection of true co-evolution within the BGC.

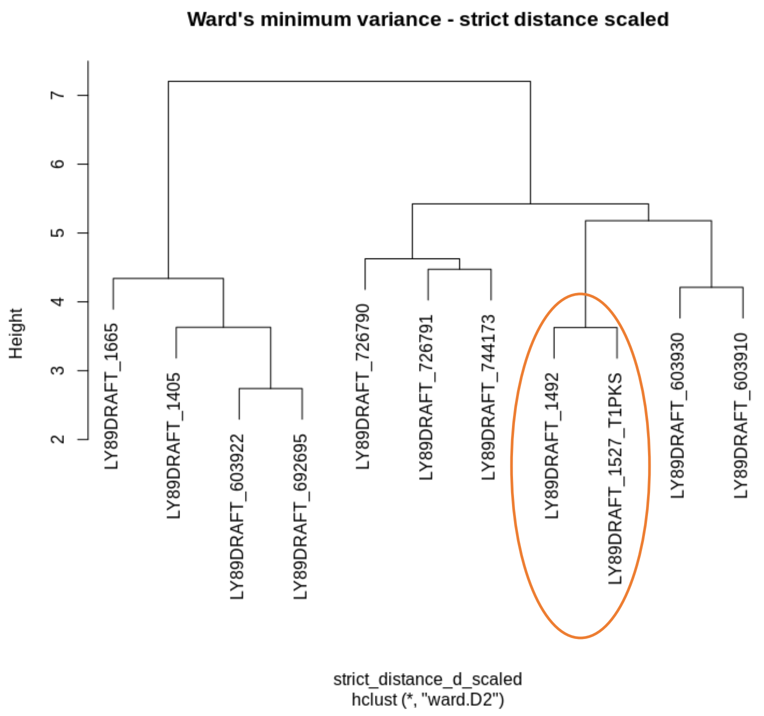

Figure 3 Standard output of the analysis of the putative T1pks BGC of *Phialocephala scopiformis* (located on scaffold NW_017263581, 125525-172708 nt). Dendrogram based on the Euclidean distances within the scaled strict distance matrix clustered using Ward´s minimum variance method aiming at finding compact spherical clusters, with the implemented squaring of the dissimilarities before cluster updating. The clustering mentioned in the text is indicated by an orange circle.

We moved on to evaluate the score plot of the first two principal components (PC) of the principal component analysis (PCA) performed on the strict distance matrix (Figure 4 A). After inspecting the explained percentage of variance from each PC (indicated as Comp 1 and Comp 2 in Figure 4), we observed a clear clustering of the core enzyme LY89DRAFT_1527 with LY89DRAFT_1492 and LY89DRAFT_603930. Whereas LY89DRAFT_603910 clearly clustered with a different group of genes. This clustering pattern is further supported by the score plot of the first two PC of the PCA performed on the combined distance matrix (Figure 4 B).

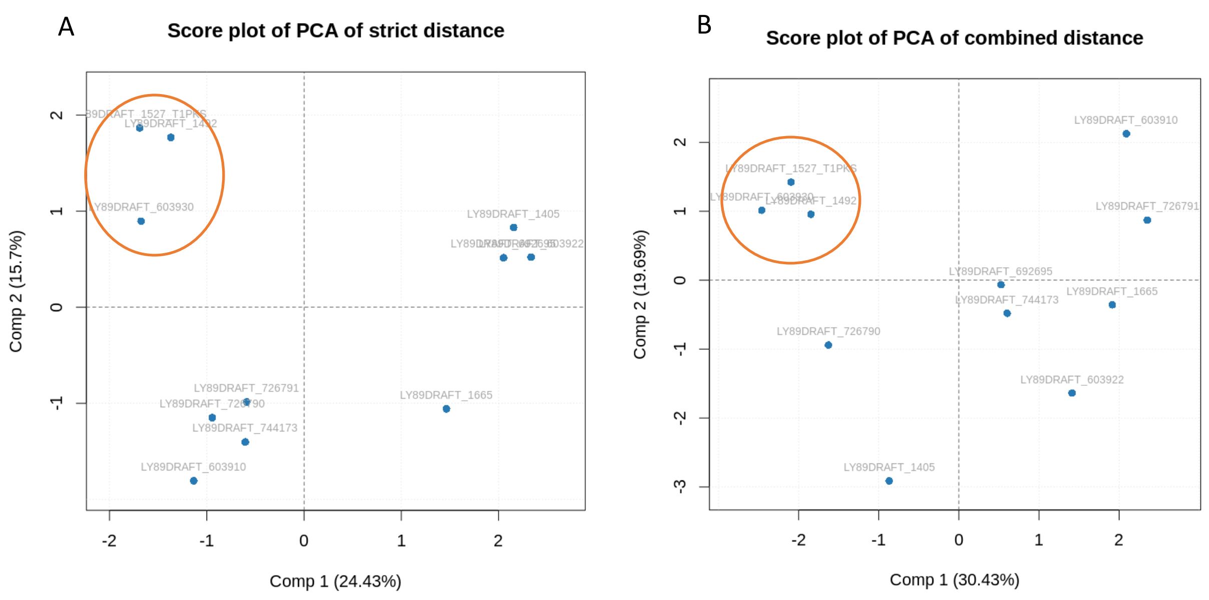

Figure 4 Standard output of the analysis of the putative T1pks BGC of *Phialocephala scopiformis* (located on scaffold NW_017263581, 125525-172708 nt). A - Score plot of the first two principal components (PC) of the principal component analysis (PCA) performed on the strict distance matrix. The clustering mentioned in the text is indicated by an orange circle. B - Score plot of the first two PC of the PCA performed on the combined distance matrix. The clustering mentioned in the text is indicated by an orange circle.

This lead to the hypothesis, that the T1pks LY89DRAFT_1527 with the putative serine hydrolase LY89DRAFT_1492 and the putative short chain dehydrogenase/reductase LY89DRAFT_603930 are responsible for the biosynthesis of the secondary metabolite (SM) encoded in this BGC, because they exhibit a shared co-evolution based on the FunOrder analysis. This hypothesis would have to be verified by corresponding *in-vitro*/*in-vivo* methods.

References:

1. Walker AK, Frasz SL, Seifert KA, Miller JD, Mondo SJ, LaButti K, et al. Full Genome of Phialocephala scopiformis DAOMC 229536, a Fungal Endophyte of Spruce Producing the Potent Anti-Insectan Compound Rugulosin. Genome Announc. 2016;4(1).
2. Blin K, Wolf T, Chevrette MG, Lu X, Schwalen CJ, Kautsar SA, et al. antiSMASH 4.0-improvements in chemistry prediction and gene cluster boundary identification. Nucleic Acids Res. 2017;45(W1):W36-W41.
3. Camacho C, Coulouris G, Avagyan V, Ma N, Papadopoulos J, Bealer K, et al. BLAST+: architecture and applications. BMC Bioinformatics. 2009;10:421-.
