# Supplementary material for: FunOrder: A robust and semi-automated method for the identification of essential biosynthetic genes through computational molecular co-evolution
Source: PLoS Comput Biol. 2021 Sep 27;17(9):e1009372. doi: 10.1371/journal.pcbi.1009372 (PMC8476034; doi:10.1371/journal.pcbi.1009372)
Supplement: S4 File — (PDF) [file pcbi.1009372.s012.pdf]

Pestalotiopsis fici biosynthetic gene cluster analysis with FunOrder

To give an example for the FunOrder analysis of an undescribed biosynthetic gene cluster (BGC), we chose a putative Non-ribosomal peptide synthetase (NRPS) BGC from *Pestalotiopsis fici* (1) (located on scaffold NW_006917091, 3350456 - 3550012 nt). This cluster was predicted with antiSMASH 4.3.0 (2) (Figure 1) and the output was directly analyzed with FunOrder.

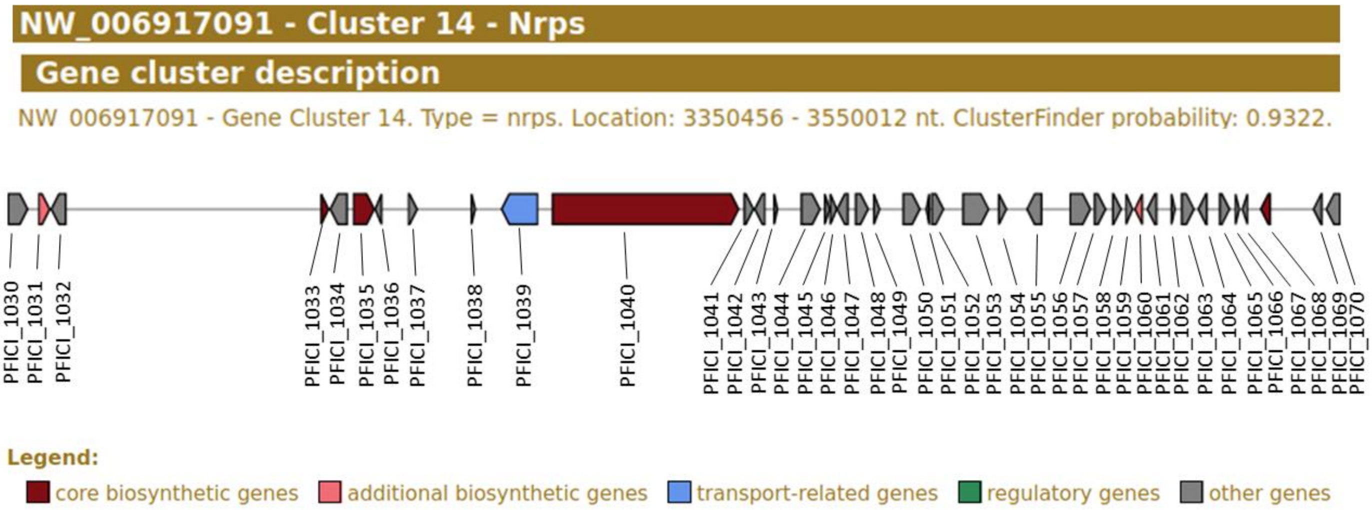

Figure 1 Screenshot of the cluster defined by antiSMASH.

The first step of the analysis was to inspect the internal co-evolutionary quotient (ICQ) calculated for this specific cluster. The ICQ was 0.5908, which is below the previously defined threshold for relevant co-evolution detected of 0.718. We therefore continued with the inspection of the heatmap based on the strict distance matrix (Figure 2). The color key in the heatmap is a direct visualization of the values of the strict distance, they are clustered based on a calculated dendrogram based on the complete linkage method. We observed a first indication of which genes might share a potential co-evolution with the core enzyme PFICI_01040 (marked as PFICI_01040_NRPS in all figures). In this case, there was no significant clustering detectable with the core enzyme PFICI_01040. PFICI_01040 appeared to share mostly high strict distances to the other genes in the BGC.

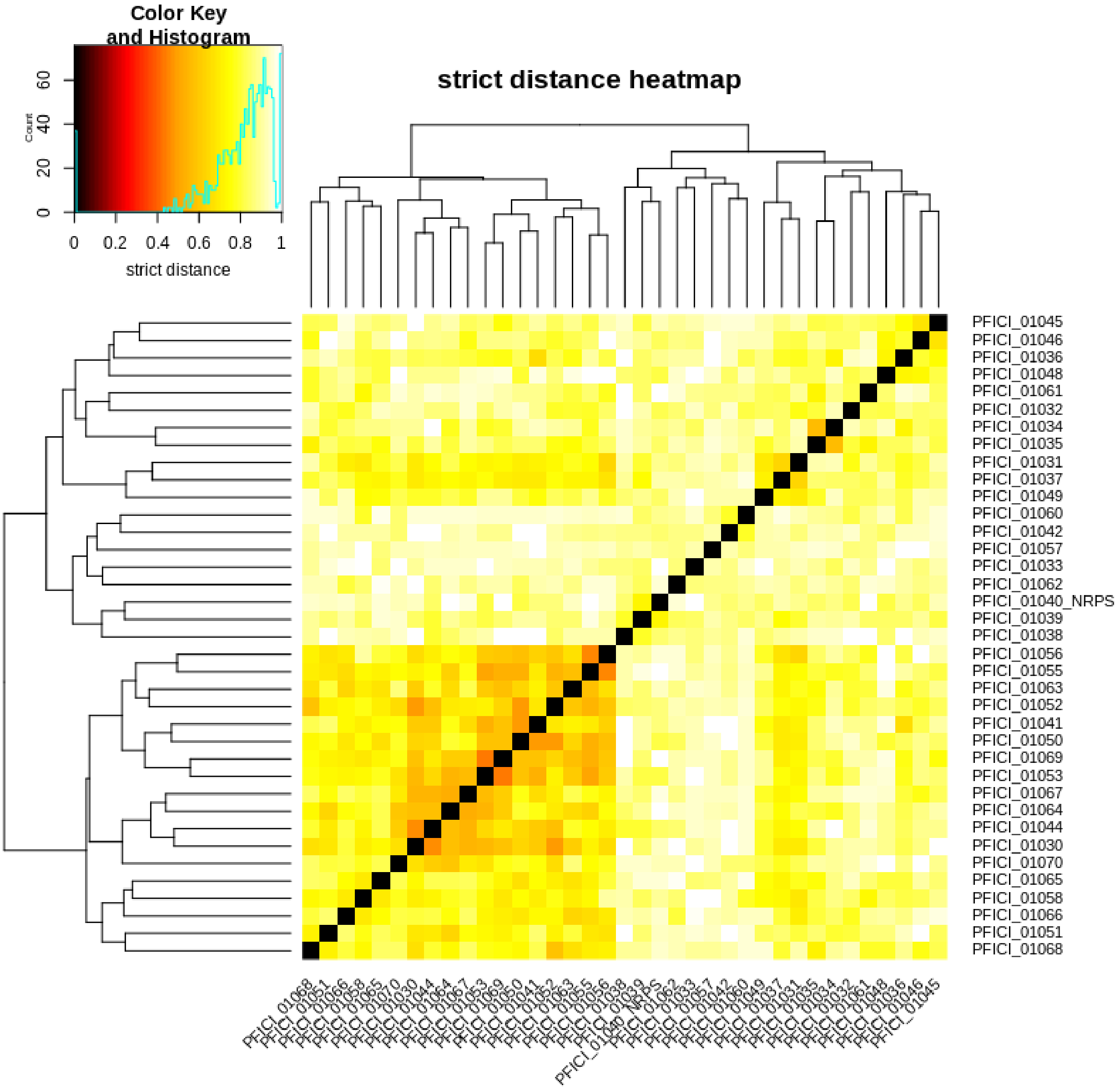

Figure 2 Standard output of the analysis of putative NRPS BGC from *Pestalotiopsis fici* (located on scaffold NW_006917091, 3350456 - 3550012 nt). Heatmap of the strict distance matrix.

Next we examined the dendrogram (Figure 3) based on the Euclidean distances within the scaled strict distance matrix clustered using Ward´s minimum variance method aiming at finding compact spherical clusters, with the implemented squaring of the dissimilarities before cluster updating. Again, we looked for the core enzyme PFICI_01040. This enabled us to determine that PFICI_01040 seems to share the strongest co-evolution within the BGC with PFICI_01039 (annotated as hypothetical protein and recognized by antiSMASH as putative multi drug transporter) and PFICI_01038 (annotated as hypothetical protein and revealed to contain a putative DNA binding domain based on a sequence similarity search using blastp (3) against the non redundant protein database and conserved domain database). This clustering considered the complete strict distance matrix, including potential noise, which could distort the detection of true co-evolution within the BGC.

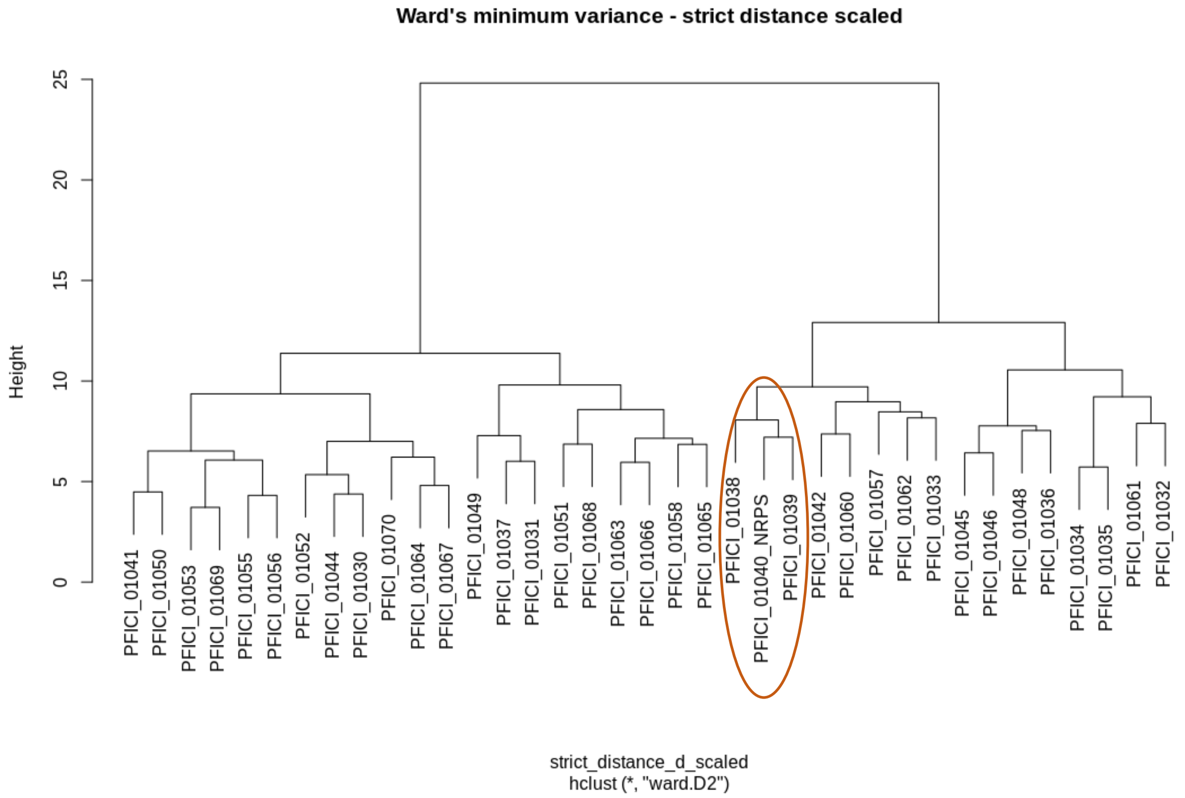

Figure 3 Standard output of the analysis of the putative NRPS BGC from *Pestalotiopsis fici* (located on scaffold NW_006917091, 3350456 - 3550012 nt). Dendrogram based on the Euclidean distances within the scaled strict distance matrix clustered using Ward´s minimum variance method aiming at finding compact spherical clusters, with the implemented squaring of the dissimilarities before cluster updating. The clustering mentioned in the text is indicated by an orange circle.

We moved on to evaluate the score plot of the first two principal components (PC) of the principal component analysis (PCA) performed on the strict distance matrix (Figure 4). After inspecting the explained percentage of variance from each PC (indicated as Comp 1 and Comp 2 in Figure 4), we observed 6 genes clustering with the core enzyme PFICI_01040 (Table 1). As non-ribosomal peptides (NRP) produced by NRPS can undergo several modifications after their synthesis (4), the number of genes clustering is no surprise.

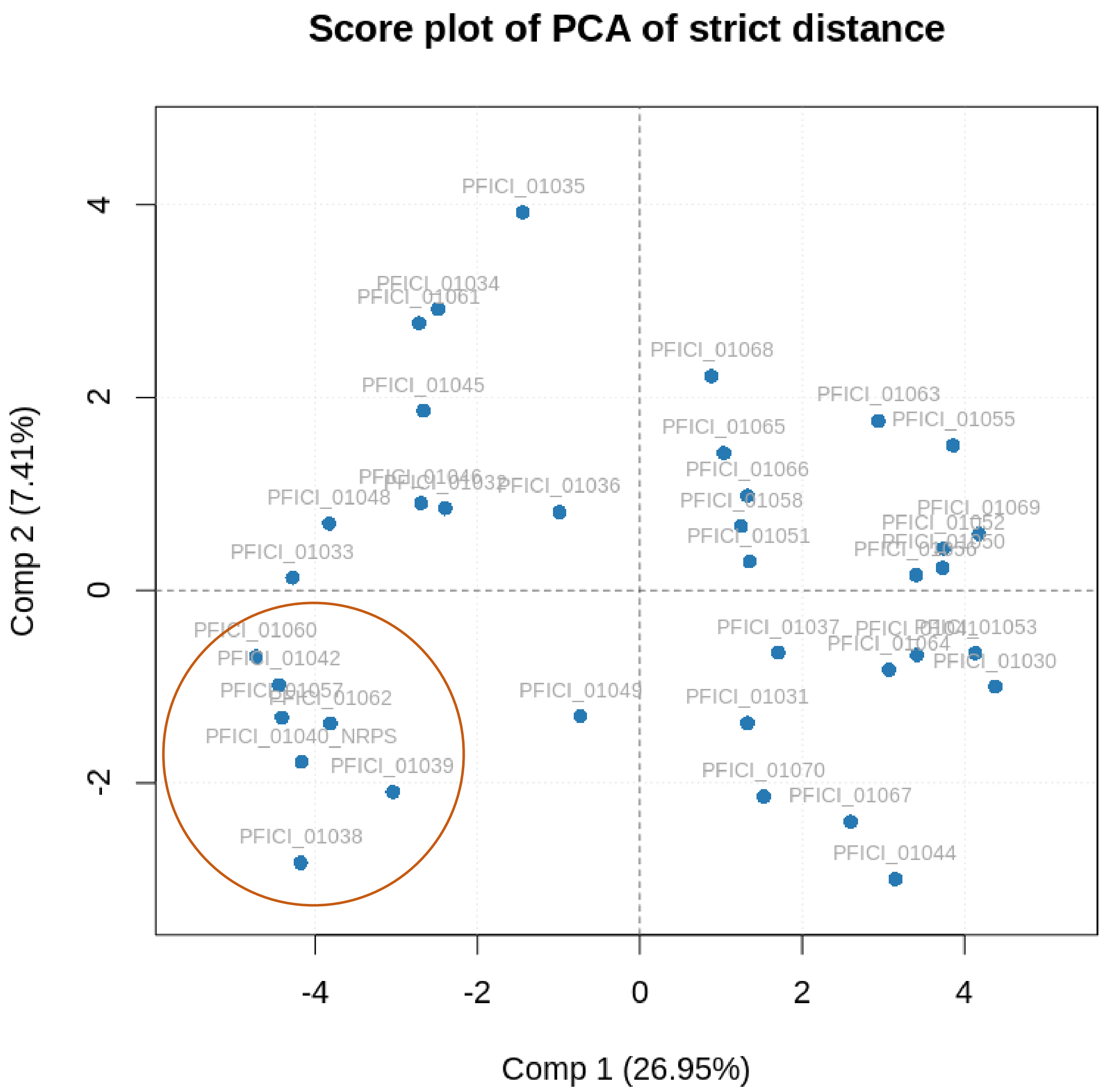

Figure 4 Standard output of the analysis of the putative NRPS BGC from *Pestalotiopsis fici* (located on scaffold NW_006917091, 3350456 - 3550012 nt). Score plot of the first two principal components (PC) of the principal component analysis (PCA) performed on the strict distance matrix. The clustering mentioned in the text is indicated by an orange circle.

Table 1 Genes clustering in the Score plot of the first two principal components (PC) of the principal component analysis (PCA) performed on the strict distance matrix with the core enzyme PFICI_01040.

**Gene Locus-tag**	**Annotation**	**Manual annotation**
PFICI_01040	Hypothetical protein	Putative NRPS
PFICI_01038	Hypothetical protein	Putative DNA binding domain containing protein
PFICI_01039	Hypothetical protein	putative multi drug transporter
PFICI_01062	Hypothetical protein	Putative Hydrophobic surface binding protein
PFICI_01057	Hypothetical protein	Putative feruloyl esterase
PFICI_01042	Hypothetical protein	Putative Peroxidase
PFICI_01060	Hypothetical protein	Putative Inositol monophosphatase

Nevertheless, this led to the hypothesis, that the NRPS PFICI_01040 produces a NRP that might be finally excreted by PFICI_01039, because they exhibit a shared co-evolution based on the FunOrder analysis. Further we hypothesized that PFICI_01038 might be involved in the regulation of the transcription of the NRPS gene. The enzymes encoded by the genes PFICI_01057 and PFICI_01042 may well play a part in the modification of the NRP. Besides, it could be possible that the NRPS already produces the final compound. This possibility was supported by the overall high strict distances shared by the core enzyme with the other genes of the BGC. These hypotheses would have to be verified by corresponding *in-vitro*/*in-vivo* methods.

References:

1. Wang X, Zhang X, Liu L, Xiang M, Wang W, Sun X, et al. Genomic and transcriptomic analysis of the endophytic fungus Pestalotiopsis fici reveals its lifestyle and high potential for synthesis of natural products. BMC Genomics. 2015;16:28.
2. Blin K, Wolf T, Chevrette MG, Lu X, Schwalen CJ, Kautsar SA, et al. antiSMASH 4.0-improvements in chemistry prediction and gene cluster boundary identification. Nucleic Acids Res. 2017;45(W1):W36-W41.
3. Camacho C, Coulouris G, Avagyan V, Ma N, Papadopoulos J, Bealer K, et al. BLAST+: architecture and applications. BMC Bioinformatics. 2009;10:421-.
4. Le Govic Y, Papon N, Le Gal S, Bouchara J-P, Vandeputte P. Non-ribosomal Peptide Synthetase Gene Clusters in the Human Pathogenic Fungus Scedosporium apiospermum. Frontiers in Microbiology. 2019;10(2062).
