# Supplementary material for: FunOrder: A robust and semi-automated method for the identification of essential biosynthetic genes through computational molecular co-evolution
Source: PLoS Comput Biol. 2021 Sep 27;17(9):e1009372. doi: 10.1371/journal.pcbi.1009372 (PMC8476034; doi:10.1371/journal.pcbi.1009372)
Supplement: S5 File — (PDF) [file pcbi.1009372.s013.pdf]

Statistical analysis of the Internal Co-evolutionary Quotient (ICQ)

All the statistical tests were performed in the R environment (1). The Shapiro-Wilk test used below was used to check for the normality of the ICQ data sets (table 1). Normality assumptions underlie outlier detection hypothesis tests. If the p-value is above the set alpha significance value (0.01) then the null hypothesis is not discarded. In other words it can be considered a normal distribution.

Table 1 Shapiro-Wilk normality tests.

**ICQ data set**	**p-value**
random GC	0.01901
BioPath	0.119
BGC	0.228
sequential GC	0.2093

Table 2 Levene's Test for Homogeneity of Variance (center = median) performed on the ICQ data sets from the analysis of the BioPath, BGCs, random GCs and sequential GCs.

	**Df**	**F value**	**Pr(>F)**
**ICQ data sets**	3	2.1335	0.09933

From the output in table 2, it can be seen that the p-value was not less than the significance level of 0.05. This means that there was no evidence to suggest that the variance is statistically significantly different for the data sets. Levene’s test is an alternative to Bartlett’s test when the data is not normally distributed.

Table 3 Computed one-way ANOVA test the analysis of variance performed on the ICQ values from the analysis of the BioPath, BGCs, random GCs and sequential GCs.

	**Df**	**Sum Sq**	**Mean Sq**	**F value**	**Pr(>F)**
**ICQ data sets**	3	1.267	0.4224	33.45	6.11e-16
**Residuals**	125	1.579	0.0126		

The output in table 3 includes the columns F value and Pr(>F) corresponding to the p-value of the test. As the p-value is less than the significance level 0.05, we could conclude that there are significant differences between the ICQ data sets in the model summary. We could therefore continue to perform an analysis of variance (ANOVA). In one-way ANOVA test, a significant p-value indicates that some of the ICQ data sets means are different, but we don’t know which pairs of the ICQ data sets are different. It is possible to perform multiple pairwise-comparison, to determine if the mean difference between specific pairs are statistically significant. As the

ANOVA test was significant, we could compute Tukey HSD (Tukey Honest Significant Differences), for performing multiple pairwise-comparison between the means of the ICQ data sets. It can be seen from the output in table 4 that only the differences are significant with an adjusted p-value lower than 0.05.

Table 4 Tukey multiple comparisons of means based on an ANOVA performed on the ICQ values from the analysis of the BioPath, BGCs, random GCs and sequential GCs with a 95% family-wise confidence level.

**comparison**	**diff**	**lwr**	**upr**	**p adj**
BioPath-BGC	-0.02870077	-0.13554943	0.07814789	0.8971149
random GC-BGC	0.2120088	0.14657762	0.27743997	**0**
sequential GC-BGC	0.05503562	-0.02116633	0.13123758	0.2416214
random GC-BioPath	0.24070957	0.14076179	0.34065734	**0**
sequential GC-BioPath	0.08373639	-0.02357184	0.19104462	0.1818826
sequential GC-random GC	-0.15697317	-0.22315216	-0.09079419	**0.0000001**

References:

1. R Core Team. (2019) R: A language and environment for statistical computing. *R Foundation for Statistical Computing*.
