# Supplementary material for: FunOrder: A robust and semi-automated method for the identification of essential biosynthetic genes through computational molecular co-evolution
Source: PLoS Comput Biol. 2021 Sep 27;17(9):e1009372. doi: 10.1371/journal.pcbi.1009372 (PMC8476034; doi:10.1371/journal.pcbi.1009372)
Supplement: S6 File — (PDF) [file pcbi.1009372.s014.pdf]

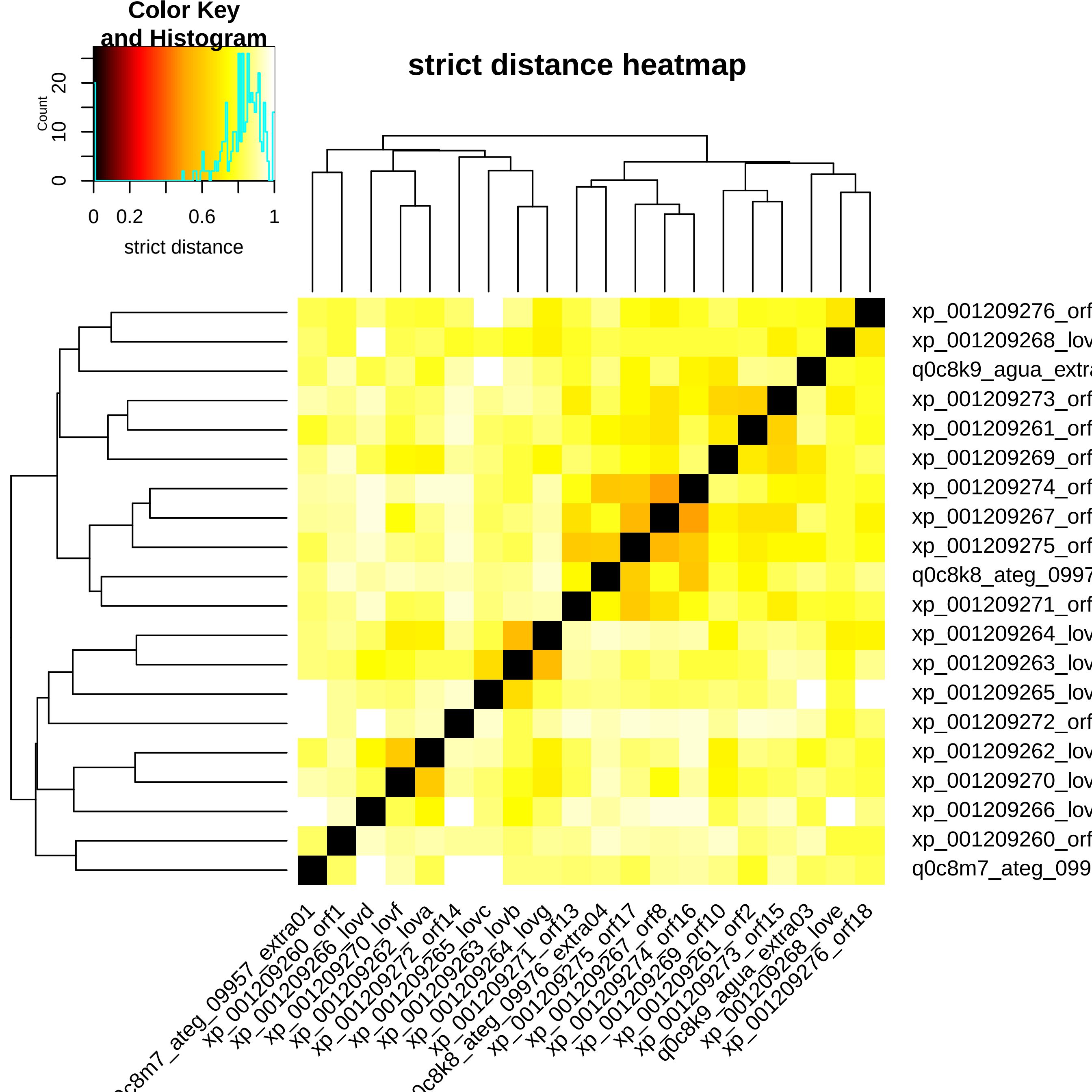

Color Key and Histogram
Count
0
10
20
0
0.2
0.6
1
strict distance
strict distance heatmap
xp_001209276_orf
xp_001209268_lov
q0c8k9_agua_extra
xp_001209273_orf
xp_001209261_orf
xp_001209269_orf
xp_001209274_orf
xp_001209267_orf
xp_001209275_orf
q0c8k8_ateg_0997
xp_001209271_orf
xp_001209264_lov
xp_001209263_lov
xp_001209265_lov
xp_001209272_orf
xp_001209262_lov
xp_001209270_lov
xp_001209266_lov
xp_001209260_orf
q0c8m7_ateg_099
c8m7_ateg_09957_extra01
xp_001209260_orf1
xp_001209266_lovd
xp_001209270_lovf
xp_001209262_lova
xp_001209272_orf14
xp_001209265_lovc
xp_001209263_lovb
xp_001209264_lovg
xp_001209271_orf13
0c8k8_ateg_09976_extra04
xp_001209275_orf17
xp_001209267_orf8
xp_001209274_orf16
xp_001209269_orf10
xp_001209261_orf2
xp_001209273_orf15
q0c8k9_agua_extra03
xp_001209268_love
xp_001209276_orf18

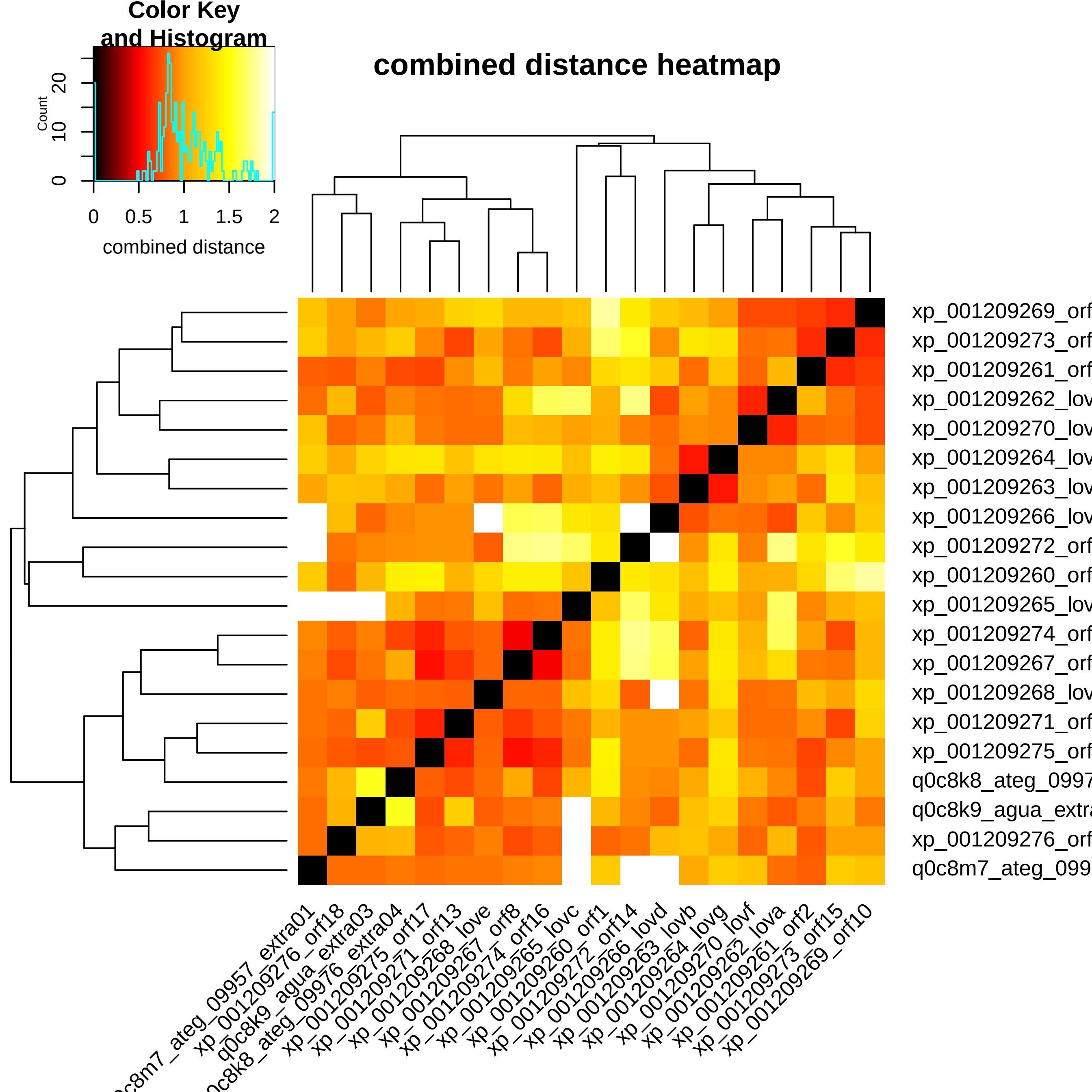

Color Key
and Histogram
Count
0
10
20
0
0.5
1
1.5
2
combined distance
combined distance heatmap
xp_001209269_orf
xp_001209273_orf
xp_001209261_orf
xp_001209262_lov
xp_001209270_lov
xp_001209264_lov
xp_001209263_lov
xp_001209266_lov
xp_001209272_orf
xp_001209260_orf
xp_001209265_lov
xp_001209274_orf
xp_001209267_orf
xp_001209268_lov
xp_001209271_orf
xp_001209275_orf
q0c8k8_ateg_0997
q0c8k9_agua_extra
xp_001209276_orf
q0c8m7_ateg_099
c8m7_ateg_09957_extra01
xp_001209276_orf18
q0c8k9_agua_extra03
0c8k8_ateg_09976_extra04
xp_001209275_orf17
xp_001209271_orf13
xp_001209268_love
xp_001209267_orf8
xp_001209274_orf16
xp_001209265_lovc
xp_001209260_orf1
xp_001209272_orf14
xp_001209266_lovd
xp_001209263_lovb
xp_001209264_lovg
xp_001209270_lovf
xp_001209262_lova
xp_001209261_orf2
xp_001209273_orf15
xp_001209269_orf10

Ward's minimum variance − strict distance scaled

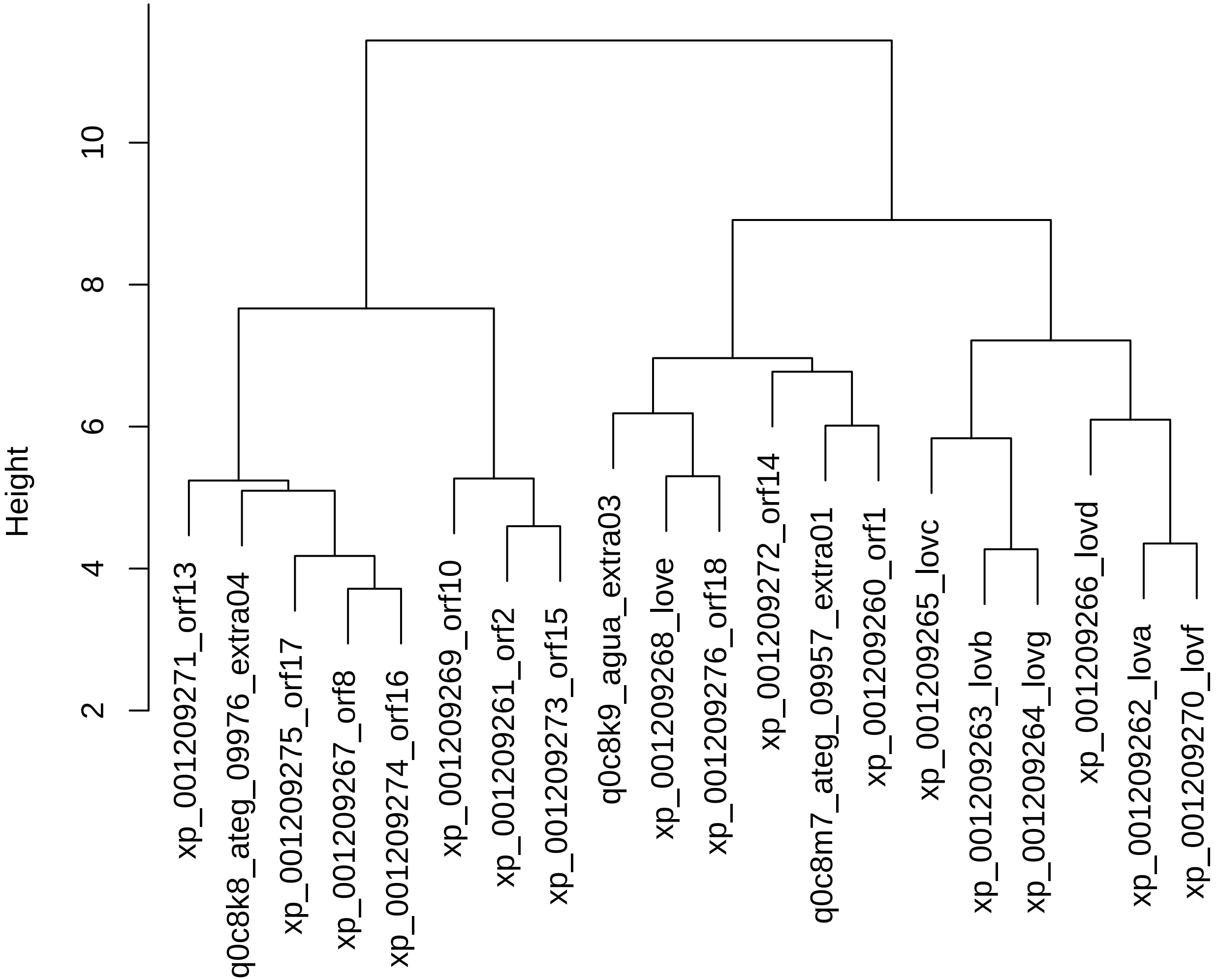

strict_distance_d_scaled
hclust (*, "ward.D2")

Ward's minimum variance − combined distance scaled

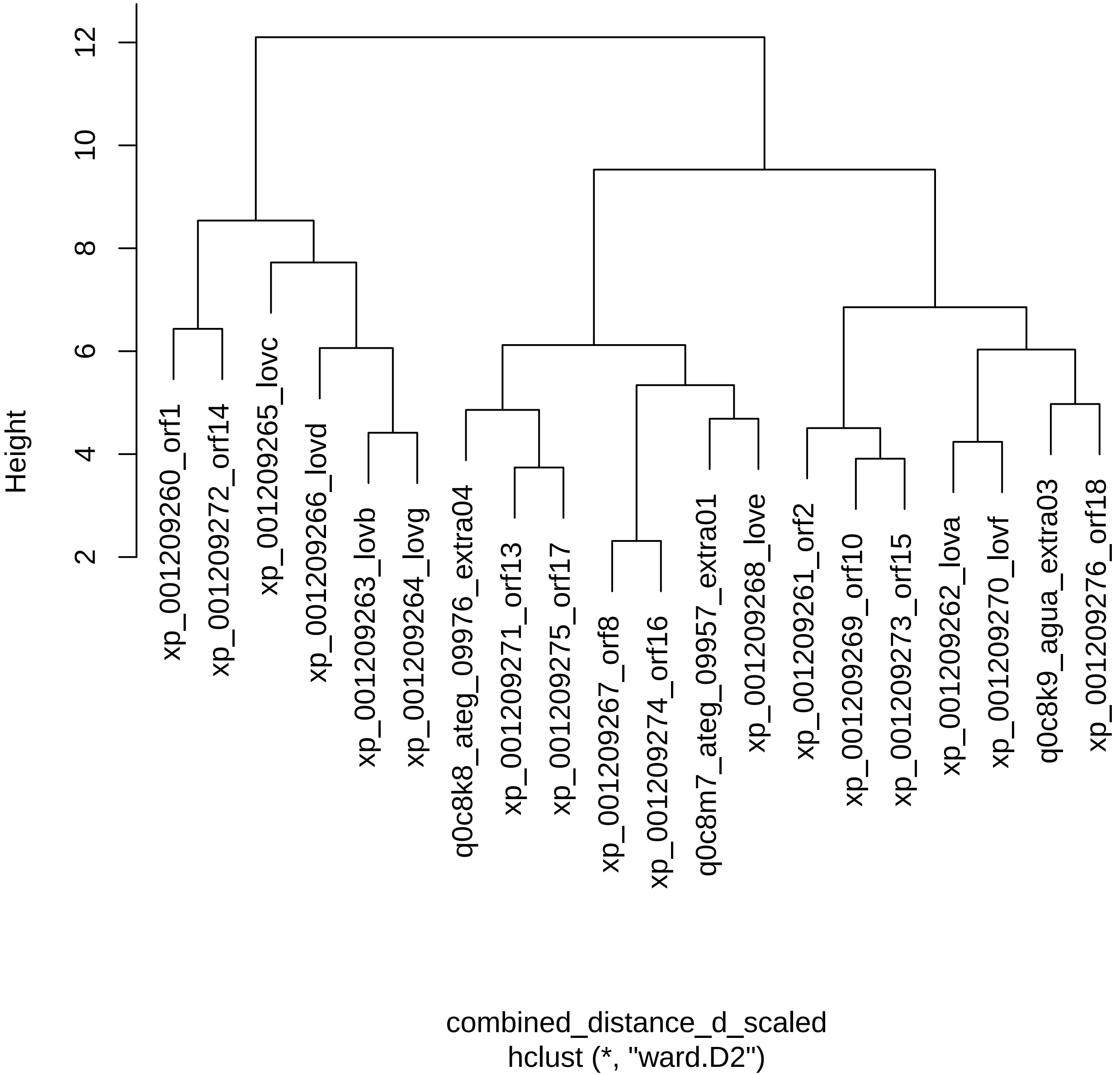

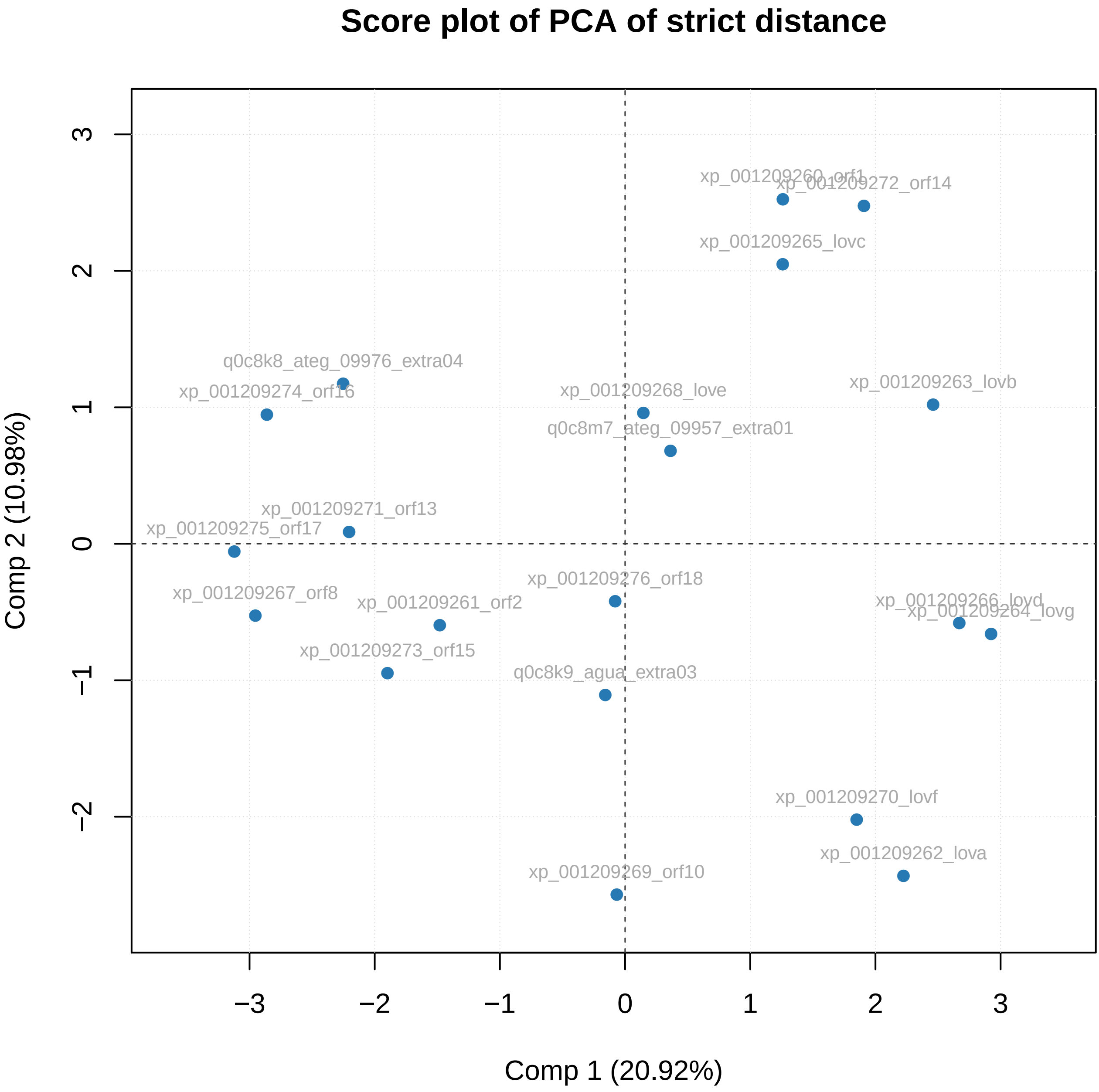

Score plot of PCA of strict distance
Comp 1 (20.92%)
Comp 2 (10.98%)
−3
−2
−1
0
1
2
3
xp_001209260_orf1
xp_001209272_orf14
xp_001209265_lovc
q0c8k8_ateg_09976_extra04
xp_001209274_orf16
xp_001209268_love
xp_001209263_lovb
q0c8m7_ateg_09957_extra01
xp_001209271_orf13
xp_001209275_orf17
xp_001209276_orf18
xp_001209267_orf8
xp_001209261_orf2
xp_001209266_lovd
xp_001209264_lovg
xp_001209273_orf15
q0c8k9_agua_extra03
xp_001209270_lovf
xp_001209262_lova
xp_001209269_orf10

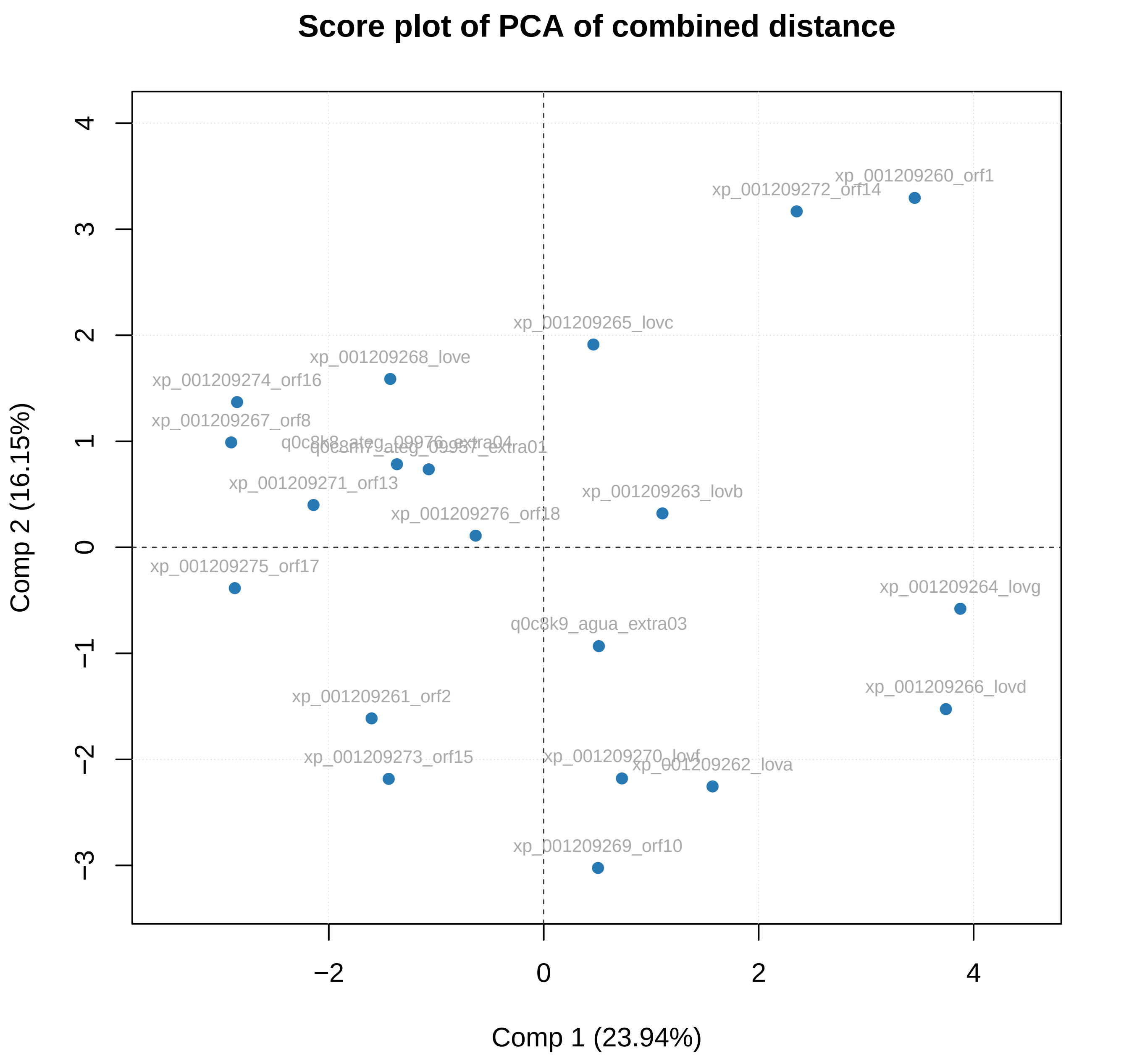
Score plot of PCA of combined distance
Comp 1 (23.94%)
Comp 2 (16.15%)
−2
0
2
4
−3
−2
−1
0
1
2
3
4
xp_001209260_orf1
xp_001209272_orf14
xp_001209265_lovc
xp_001209268_love
xp_001209274_orf16
xp_001209267_orf8
q0c8k8_ateg_09976_extra04
q0c8m7_ateg_09937_extra01
xp_001209271_orf13
xp_001209263_lovb
xp_001209276_orf18
xp_001209275_orf17
xp_001209264_lovg
q0c8k9_agua_extra03
xp_001209266_lovd
xp_001209261_orf2
xp_001209273_orf15
xp_001209270_lovf
xp_001209262_lova
xp_001209269_orf10
